# The DNA barcode reveals cryptic diversity and a new record for the genus *Leporinus* (Characiformes, Anostomidae) in the hydrographic basins of central northern Brazil

**DOI:** 10.7717/peerj.15184

**Published:** 2023-05-25

**Authors:** Maria Histelle Sousa Nascimento, Deborah Gaído Aragão, Jordânia Leticia Nascimento Silva, Renato Correia Lima, José Luis Olivan Birindelli, Elmary Costa Fraga, Maria Claudene Barros

**Affiliations:** 1 Department of Chemistry and Biology, Maranhão State University, Caxias, Maranhão, Brazil; 2 Graduate Network Program in the Biodiversity and Biotechnology of Legal Amazonia, Biological Sciences Institute, Belem, Pará, Brazil; 3 Graduate Program in Genetics, Conservation, and Evolutionary Biology, National Amazonian Research Institute, Manaus, Amazonas, Brazil; 4 Department of Animal and Plant Biology, Londrina State University, Londrina, Paraná, Brazil

**Keywords:** Molecular identification, Freshwater fish, Leporinus, Neotropical biodiversity, Systematics

## Abstract

*Leporinus* is one of the most speciose genera of the order Characiformes, with 81 valid species distributed throughout much of Central and South America. The considerable diversity of this genus has generated extensive debate on its classification and internal arrangement. In the present study, we investigated the species diversity of the genus *Leporinus* in central northern Brazil, and conclude that six valid species—*Leporinus maculatus*, *Leporinus unitaeniatus*, *Leporinus affinis*, *Leporinus venerei*, *Leporinus* cf. *friderici*, and *Leporinus piau*—are found in the hydrographic basins of the Brazilian states of Maranhão, Piauí, and Tocantins. We analyzed 182 sequences of the Cytochrome Oxidase subunit I gene, of which, 157 were obtained from *Leporinus* specimens collected from the basins of the Itapecuru, Mearim, Turiaçu, Pericumã, Periá, Preguiças, Parnaíba, and Tocantins rivers. The species delimitation analyses, based on the ABGD, ASAP, mPTP, bPTP, and GMYC methods, revealed the presence of four distinct molecular operational taxonomic units (MOTUs), identified as *L. maculatus*, *L. unitaeniatus*, *L. affinis*, and *L. piau* (from the Parnaíba River). The bPTP method restricted *L. venerei* to a single MOTU, and confirmed the occurrence of this species in the rivers of Maranhão for the first time. The separation of *L.* cf.* friderici* into two clades and the subsequent formation of different operational taxonomic units was consistent with polyphyly in this species, which indicates the existence of cryptic diversity. The arrangement of *L.* cf.* friderici* and *L. piau* in two different clades supports the conclusion that the *L. piau* specimens from Maranhão were misidentified, based on their morphological traits, reflecting the taxonomic inconsistencies that exist among morphologically similar species. Overall, then, the species delimitation methods employed in the present study indicated the presence of six MOTUs—*L. maculatus*, *L. unitaenitus*, *L. affinis*, *L.* cf. *friderici*, *L. venerei*, and *L. piau*. In the case of two other MOTUs identified in the present study, one (*L. venerei*) is a new record for the state of Maranhão, and we believe that the other represents a population of *L. piau* from the basin of the Parnaíba River.

## Introduction

The family Anostomidae is a prominent group of Neotropical fish that includes 15 genera and approximately 151 valid species (Ramirez et al., 2016; Britski & Birindelli, 2019; Ramirez et al., 2020). The most speciose genus is *Leporinus*, which has approximately 81 valid nominal species (Fricke, Eschmeyer & Laan, 2021). Géry (1977) concluded that *Leporinus* is one of the most diverse genera of the order Characiformes, which is distributed between Central America and southern South America.

The considerable diversity found in the genus *Leporinus* has led to numerous attempts to classify its species and determine its internal arrangement. A number of studies have proposed subdivisions based on the position of the mouth, and the shape and arrangement of the teeth (Borodin, 1929; Myers, 1950; Garavello, 1979). Britski & Garavello (1978) divided the genus into three groups based on coloration patterns, that is, banding, spots, and longitudinal lines, although these proposals have been contradicted by more comprehensive studies, such as those of Sidlauskas & Vari (2008) and Ramirez et al. (2016). In their cytogenetic study, Galetti, Lima & Venere (1995) confirmed the existence of a well-defined ZZ/ZW sex chromosome system in six *Leporinus* species. These authors proposed that the presence of the ZW system represents a synapomorphy, and that the six species with this system form a monophyletic group. This conclusion is reinforced by morphological traits, such as coloration patterns, relatively large body sizes, and the number of teeth, as confirmed by Ramirez et al. (2016), which led to the allocation of this group to a new genus, *Megaleporinus*, by Ramirez, Birindelli & Galetti (2017).

Using osteological markers, Sidlauskas & Vari (2008) evaluated the phylogenetic relationships of the anostomids, and concluded that this family is monophyletic, although they were unable to confirm the monophyly of the genus *Leporinus*. Ramirez et al. (2016) used nuclear and mitochondrial molecular markers to confirm the paraphyly of the genus *Leporinus*, and concluded that the recuperation of the monophyly of the group would depend on further taxonomic reviews, including the creation of new genera and the description of new species.

Traditional taxonomic approaches have been essential for the delimitation of anastomid species based on morphological traits, although this does not necessarily resolve some natural groups, given that morphologically similar species may be assigned to the same nominal taxon (Bickford et al., 2007). Deciphering and defining cryptic diversity accurately is fundamental to the understanding of the ecological, biogeographic, and evolutionary patterns of a group of organisms, in addition to its other biological features (Kress et al., 2015).

Hebert et al. (2003) proposed the use of a DNA barcode, based on a standard sequence of the mitochondrial Cytochrome Oxidase subunit I (COI) gene, as the basis for a global species identification system. This approach has been widely-used for the identification of species and the resolution of cryptic diversity within genera and, in particular, in species complexes. A species complex consists of a group of closely-related taxa that have typically undergone recent speciation, which means that their taxonomic differences are still incipient, as observed in the case of the *Leporinus* cf. *friderici* species complex, in which Silva-Santos et al. (2018) confirmed the presence of eight distinct Molecular Operational Taxonomic Units (MOTUs) arranged in three clades.

*Leporinus* is not only one of the most diverse fish genera, but its species also play an important ecological role in many freshwater ecosystems, as well as having considerable economic and social importance for local fisheries. Given this, we compiled a dataset of the mitochondrial COI gene of 182,179 *Leporinus* specimens, which included specimens from the hydrographic basins of the Brazilian state of Maranhão to verify the potential intrageneric diversity of this genus, *i.e.,* the presence of different putative species for the study region.

Here we present the diversity of *Leporinus* from hydrographic basins of central northern Brazil. We used integrative taxonomy tools to assess the species diversity of *Leporinus* based on (i) morphological identification from external characters, (ii) morphological identification from dentary characters, and (iii) molecular identification from COI gene fragment.

## Material and Methods

### Sampling

**Figure 1:**
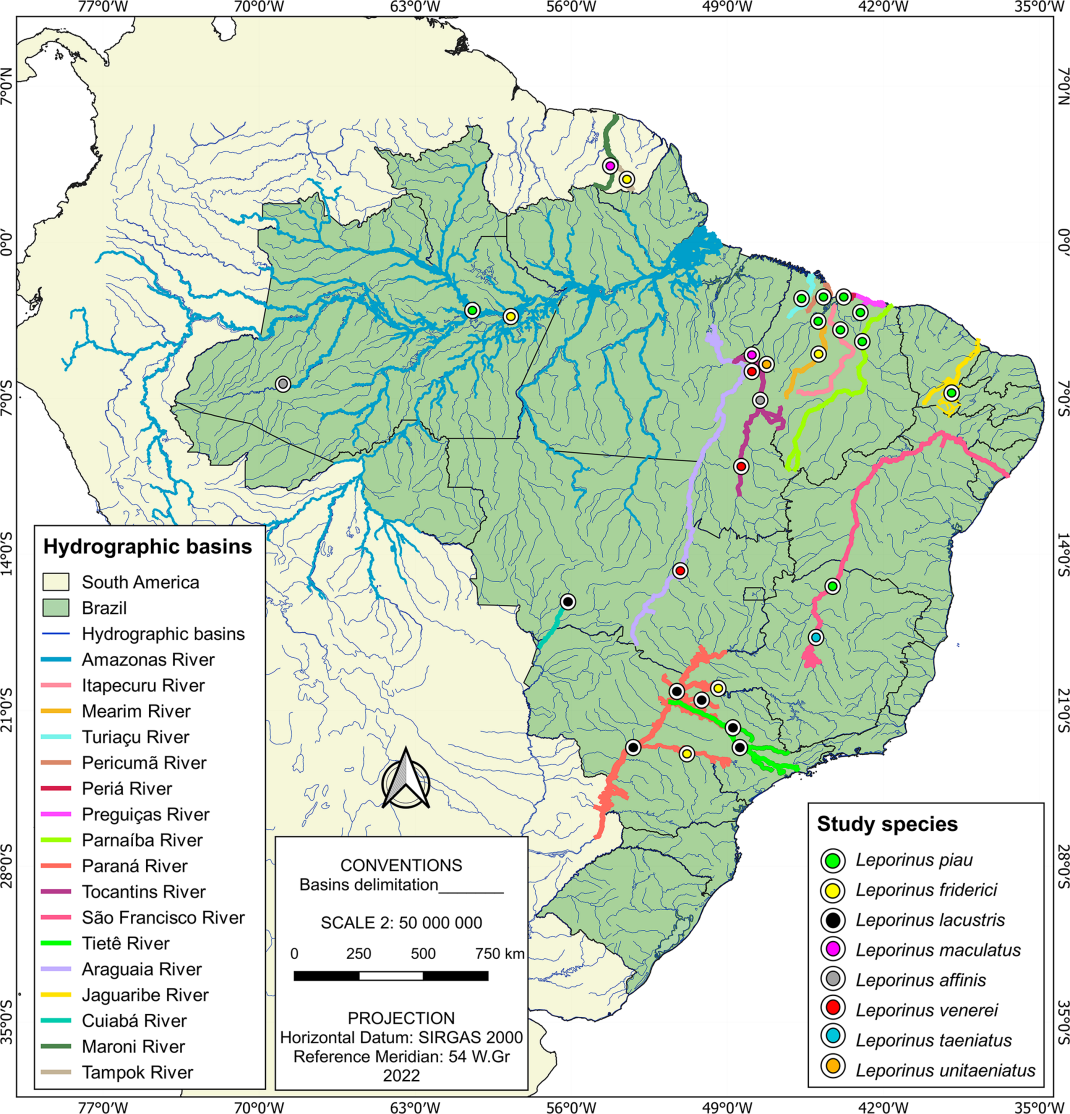
Sample localities. Each data point indicates the location where Leporinus samples were collected.

The present study was based on the analysis of a total of 185 sequences, of which 182 were of *Leporinus* species, with the other three representing the outgroup. The vast majority (157) of these 182 *Leporinus* sequences were collected during the present study, being extracted from specimens collected from basins in the Brazilian states of Maranhão (Itapecuru, Mearim, Turiaçu, Pericumã, and Periá rivers), Piauí (Parnaíba River), and Tocantins, that is, the Tocantins River (Fig. 1 and Table S1). The other 25 sequences were obtained from GenBank (Table S2).

The samples from the rivers of Maranhão, Piaui and Tocantins were obtained during extensive fieldwork, which has been ongoing since 2006. This research was authorized by the Brazilian Institute of the Environment and Renewable Natural Resources (IBAMA) through license 02012.004159/2006, and licenses ICMBio/MMA 42119-1/2013, ICMBio/MMA 46367-1/2015, ICMBio/MMA 83138-1/2022, ICMBio/MMA 73790-6/2022 issued by the Chico Mendes Institute for Biodiversity Conservation.

After collection, the specimens were taken to the Genetics and Molecular Biology Laboratory (GENBIMOL) of the Advanced Studies Center of Maranhão State University (CESC/UEMA), where they photographed and registered using a coding system. Samples of muscle tissue were extracted from the specimens for the genetic analyses. The specimens were then fixed em 10% formaldehyde and conserved in 70% alcohol, before being sent to the Museum of Zoology at Londrina State University (MZUEL) in Londrina, Paraná, Brazil, for morphological identification and cataloguing. The study of wild animals was approved by the Regulatory Committee for the Ethical Treatment of Animals of Maranhão State University (protocol 47/2022) and by the Committee for the Ethical Use of Animals of the National Institute for Amazonian Research, registered under protocol number 006/2021, SEI 01280.000116/2021-45.

The total DNA was extracted using the Wizard Genomic DNA Purification kit from Promega, following the maker’s instructions. The genomic region was isolated and amplified by Polymerase Chain Reaction (PCR), using the universal primers COI FishF1 5′-TCAACCAACCACAAAGACATTGCCAC-3′ and COI FishR1 5′- TAGACTTCTGGGTGGCCAAAGAATCA-3′, described by Ward et al. (2005). The samples were sequenced by the Sanger, Nicklen & Coulson (1977) method, using the Big Dye kit in an ABI Prism™ 3500 automatic sequencer (Applied Biosystems, EUA).

The sequences were aligned and edited in the Clustal W (Thompson, Higgins & Gibson, 1994) application of the Bioedit 7.2.5 program (Hall, 1999). All newly generated sequences (175) were deposited in GenBank under accession numbers OP781850–OP781884, OP782222 –OP782283, OP782350–OP7882375 and OP782385–OP782418 (Table S1). The haplotypes were delineated in DnaSP 5.1 (Librado & Rozas, 2009). The mean genetic distances and the Maximum Likelihood (ML) tree were obtained in MEGA X (Kumar et al., 2018), using the Kimura 2-Parameter and Hasegawa-Kishino-Yano (HKY) models, respectively, with the trees being reconstructed using 1,000 bootstrap replicates.

The optimum evolutionary model for the construction of the Bayesian Inference (BI) and Maximum Likelihood (ML) trees was generated in JModelTest2 (Darriba et al., 2012), which is available at CIPRES Science Gateway v3.3 (Miller, Pfeiffer & Schwartz, 2010), using the Hasegawa-Kishino-Yano (HKY+G+I) algorithm. The BI tree was generated in BEAST v.1.10.4 (Drummond et al., 2012; Suchard et al., 2018), using the relaxed lognormal clock (Drummond et al., 2006) and the birth-death speciation model (Gernhard, 2008).

This analysis was based on 40,000,000 generations with the log files being verified in Tracer v1.6 (Rambaut et al., 2014) to evaluate convergence and the most adequate burn-in, with the convergence being considered adequate when the Effective Sample Size (ESS) was over 200. The trees generated in BEAST were summarized in TreeAnnotator v.10.4 (Suchard et al., 2018) to obtain the consensus tree, which was then visualized and edited in Fig Tree v1.4.2 (Rambaut, 2014) and the Inkscape image editing system. Clades with a bootstrap percentage of at least 85% or posterior probability of at least 0.95 were considered to be well supported.

The delimitation analyses of the MOTUs of the COI gene were run using the following models: the Automatic Barcode Gap Discovery (ABGD), Assemble Species by Automatic Partitioning (ASAP), Poisson Tree Process (PTP), and the Generalized Mixed Yule Coalescent (GMYC) model. The ABGD test (Puillandre et al., 2012) was run in https://bioinfo.mnhn.fr/abi/public/abgd/ using the dataset of aligned sequences, while the ASAP test (Puillandre, Brouillet & Achaz, 2020) was implemented in https://bioinfo.mnhn.fr/abi/public/asap/asapweb.html using the matrix of genetic distances, extracted using MEGA X, as the input. The PTP (Zhang et al., 2013) was run on the web server https://species.h-its.org/. In this case, the input was the Maximum Likelihood phylogenetic tree produced in RaxML v.8.29 (Stamatakis, 2014), which is available in the CIPRES Science Gateway v3.3 (Miller, Pfeiffer & Schwartz, 2010). The GMYC (Fujisawa & Barraclough, 2013) was based on the ultrametric consensus tree constructed in BEAST v1.10.1, which was processed in the Ape (Paradis & Schliep, 2019), Splits (Ezard, Fujisawa & Barraclough, 2009), Paran (Dinno, 2009), and Mass (Venables & Ripley, 2002) packages available in the R *v.* 4.1.0 software (R Core Team, 2021).

## Results

**Figure 2:**
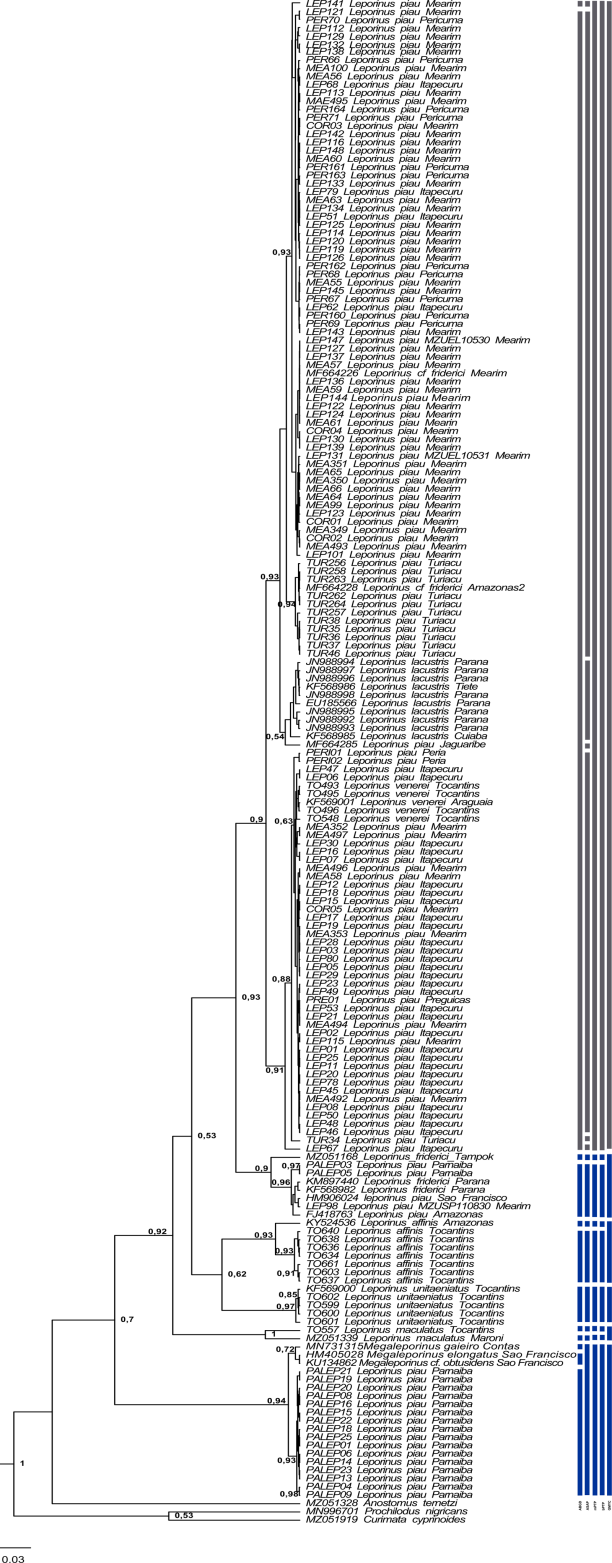
Bayesian Inference tree showing the arrangement of the MOTUs of the Leporinus species analyzed in the present study. This arrangement was obtained using the ABGD, ASAP, mPTP, bPTP, and GMYC species delimitation approaches for the analysis of the mitochondrial COI gene, based on the Hasegawa-Kishino-Yano (HKY+G+I) algorithm, generated in BEAST. The species delimitated by the specific estimates are shown by the vertical bars, with the color representing the current status of the species. The blue bars correspond to valid species, while the gray bars indicate the species delimited diûerently from the current classification.

**Figure 3:**
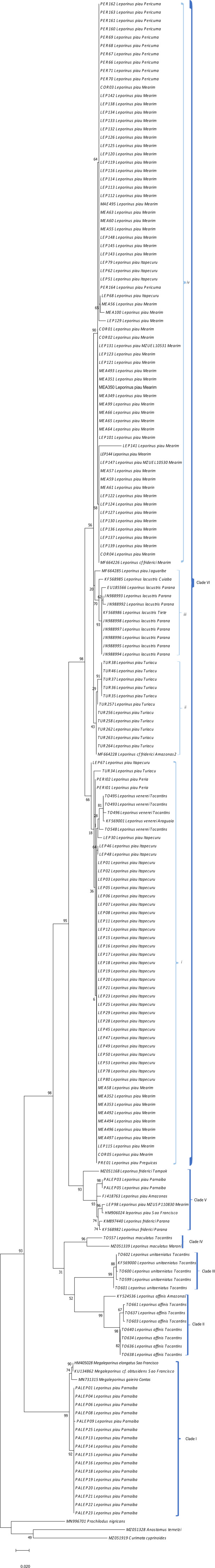
Maximum Likelihood tree of the Leporinus species. Maximum Likelihood tree showing the arrangement of the Leporinus species based on the analysis of 185 samples of the mitochondrial COI gene using the Hasegawa-Kishino Yano (HKY+G+I) algorithm, generated in MEGA X. The node support, that is, is given by the Bayesian posterior probability/ML bootstrap values, respectively. Each clade and its subdivisions (when present) are demarcated by the brackets. The Roman numerals in upper case represent the clades, while those in lower case indicate the subclades.

**Figure 4:**
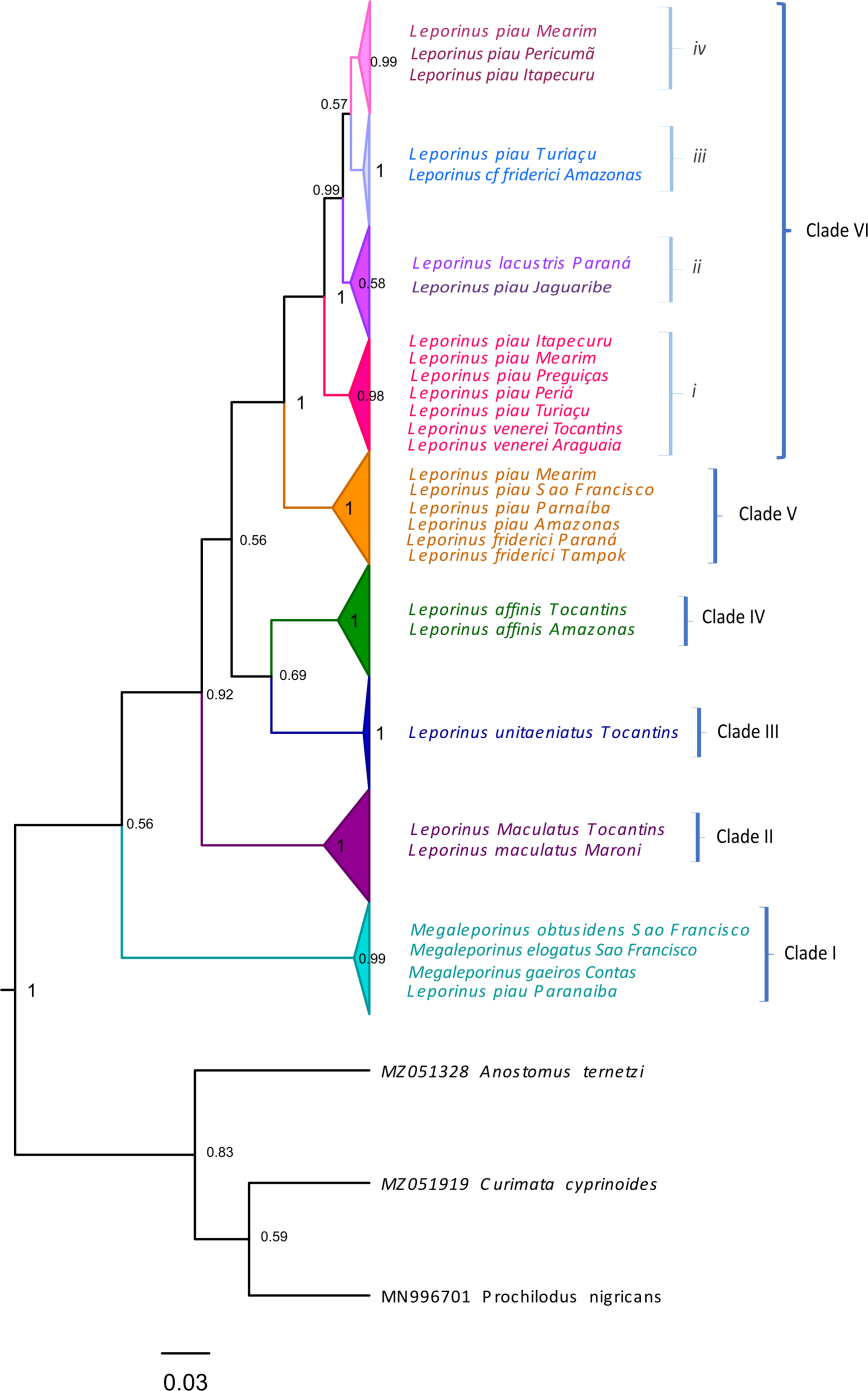
Collapsed Bayesian inference tree of the MOTUs of the Leporinus species. Collapsed Bayesian inference tree showing the arrangement of the MOTUs of the Leporinus species based on 185 samples of the mitochondrial COI gene analyzed using the Hasegawa-Kishino-Yano (HKY+G+I) algorithm, applied in BEAST. The groups were deûned by observing the congruence between the MOTUs generated in the species delimitation analyses based on the ABGD, ASAP, mPTP, bPTP, and GMYC methods.

The present study focused on 182 sequences of the COI gene of *Leporinus*, each consisting of 620 base pairs (bps). The phylogenetic trees generated by the ML and BI analyses were highly congruent and well-supported at both the intra- and interspecific levels (Figs. 2–4), except in the case of *Leporinus piau*, which grouped with either *Leporinus* cf. *friderici* or *Leporinus venerei*. The ABGD analysis delimited 12 MOTUs, while the ASAP defined 15, the mPTP and bPTP each delimited nine, and the GMYC, six MOTUs (Fig. 2).

The results of the five delimitation methods applied in the present study had three species in common—*L. maculatus*, *L. unitaeniatus*, and *L. affinis*—as well as differentiating two specimens (PALEP01 and PALEP09) from the basin of the Parnaíba River in a distinct molecular taxonomic unit, which indicates the occurrence of a fourth species, which we believe to be *L. piau*.

In the present study, the five delimitation methods had three species in common—*L. maculatus*, *L. unitaeniatus*, and *L. affinis*—and differentiated specimens from the basin of the Parnaíba River in a distinct molecular taxonomic unit, which is more basal than the other *Leporinus* species, and groups with the the *Megaleporinus* species that were previously assigned to *Leporinus*.

Clade VI (Fig. 3) was strongly supported, and includes *L. venerei*, *L. lacustris*, *L. piau*, and *L.* cf*. friderici*, with *L. piau* occurring in Maranhão, in the Mearim, Itapecuru, Pericumã, Turiaçu, Preguiças and Periá basins. In this case, the clade was formed by *L. venerei* from the Tocantins basin, *L. lacustris* from the basin of the Paraná River, *L. piau* from the Jaguaribe, Itapecuru, Mearim, Pericumã, Turiaçu, Preguiças and Periá basins, and *L.* cf. *friderici* from the Amazon and Mearim basins, which all share a single molecular taxonomic unit. Only the bPTP analysis separated *L. venerei* from *L. lacustris*, *L. piau*, and *L.* cf*. friderici*, which together formed a single MOTU in the ABGD, ASAP, mPTP, and GMYC models (Fig. 2).

The BI and ML analyses identified the formation of subclades within clade VI (Figs. 2 and 3), in which the *L. piau* from Maranhão, in the Itapecuru, Turiaçu, Mearim, and Periá basins, grouped with *L. venerei* from the Tocantins basin, with genetic distances ranging from only 0.16% to 1.54% (Table S3). Other *L. lacustris* and *L. piau* subclades were identified in the Jaguaribe basin, where the genetic distances ranged from 0.0% to 3.5% (Table S3). The *L. piau* subclade from Maranhão, found in the Itapecuru, Mearim, Pericumã, and Turiaçu basins, grouped with *L.* cf*. friderici* from the Mearim (Maranhão) and Amazon basins (Amazonas state), with genetic distances of between 0.16% and 5.88% (Table S3). All three groups were supported by significant posterior probability (BI) and bootstrap(ML) values (Figs. 2 and 3).

*Leporinus* cf. *friderici*, whose type locality is the basin of the Tampok River in French Guiana, formed a group together with *L. piau* from the basins of the São Francisco, Amazon, and Mearim rivers, an arrangement found in both the species delimitation models and the BI and ML trees. In the ABGD, ASAP mPTP, and bPTP delimitation models, however, *L.* cf. *friderici* was differentiated in its own operational unit (Fig. 2).

The genetic distance matrix derived from the molecular taxonomic units revealed relatively high values for both the intra- and inter-MOTU distances. The highest mean intra-MOTU distance was 5.9%, in *L. maculatus*, while the lowest mean was 0.4%, in *L. unitaeniatus*, whereas the mean inter-MOTU distances ranged from 7.8% to 17.4% The MOTUs formed by *L. venerei*, *L. lacustris*, *L. piau*, and *L.* cf. *friderici* were separated by a mean genetic distance of 2.2% (Table 1). In this context, it is important to note the genetic distance of 7.8% between *L. piau* (MOTU 1) and *L.* cf. *friderici* (MOTU 2), which may be the result of an error in the identification of the species of one of the groups.

Given the levels of congruence identified in the different delimitation analyses applied in the present study, the ASAP method appeared to be the most effective interpretation, in biological terms, of the dataset considered here, given that it identified 10 MOTUs, which distinguished four of the seven nominal species, including *L. venerei*, in a distinct MOTU. This confirmed the occurrence of this species the Itapecuru, Mearim, Turiaçu, Preguiças and Periá basins, which constitutes the first record of *L. venerei* in the Brazilian state of Maranhão.

## Discussion

An adequate taxonomic assessment is fundamental for the success of many types of biological research, and DNA data have provided additional insights for the resolution of taxonomic questions in many groups of organisms, including elements of the megadiverse Neotropical fish fauna, such as the anostomids. The COI barcode proved to be an extremely valuable tool for the identification and separation of the species assessed in the present study, based on the analysis of genetic distances and species delimitation, which identified evidence of the potential presence of more than one taxon in some nominal species.

In many previous studies of DNA barcoding and molecular diversity, the number of species or lineages delimited by the analysis has tended to exceed the number of nominal taxa or even the morphospecies analyzed (Carvalho et al., 2018). A similar tendency was observed here, in addition to the opposite pattern, given that, in some species delimitation analyses, more than one valid species was allocated to the same MOTU, as in the case of *L. venerei*, *L. lacustris*, *L.* cf. *friderici*, and *L. piau*.

In the present study, the *L. venerei*, *L. lacustris*, *L.* cf. *friderici*, and *L. piau* specimens were assigned to a single molecular taxonomic unit by the ABGD, ASAP, mPTP, and GMYC methods, reflecting their similar morphological characteristics, such as their coloration pattern, dental formula, and meristic parameters (Table 2), although the intra-MOTU analyses revealed mean genetic distances of 2.7% (Table 1), ranging from 0.0% to 6.11% (Table S3). This is consistent with the current classification of the valid nominal species (BI and ML analyses: Figs. 2–4; Table 1). All the species of clade VI shared the same morphological pattern, which is considered to be diagnostic of *L. friderici*, such as the number of spots along the lateral line (1–3) and the 4/4 dental formula, except for *L. venerei*, which has four teeth in the pre-maxilla and three in the dentary row. *Leporinus lacustris* and *L. venerei* are highly similar morphologically, given their relatively tall body, terminal mouth, long, dark anal fin, and three spots on the lateral line (Britski & Birindelli, 2008; Silva-Santos et al., 2018). *Leporinus* piau presents the D-type coloration pattern described by Garavello (1979), which consists of three well-defined black spots on the lateral line, and four teeth in both rows, with a dental formula of 4/4. The *L. venerei*, *L. lacustris*, *L.* cf. *friderici*, and *L. piau* MOTU was subdivided into three subclades (*i*, *ii*, *iii* and *iv*—Fig. 3). Subclade *i* includes *L. piau* from Maranhão and *L. venerei* from the Tocantins basin, while subclade *ii* has *L.* cf. *friderici* from the Amazon and *L. piau* from the Turiaçu basins. Subclade *iii* groups *L. lacustris* and *L. piau* from the Jaguaribe basin, and subclade *iv* groups *L. piau* from Maranhão and *L.* cf. *friderici* from the Mearim. The composition of subclade *i* (*L. piau* and *L. venerei*—Fig. 3), together with the diagnostic morphological features of the species, indicates that the specimens from the basins of Maranhão identified as *L. piau* may in fact be *L. venerei*, which would be the first record of this species from this Brazilian state.

**Table 1:** Mean genetic distance of the Leporinus and Megaleporinus. Mean genetic distance, based on the Kimura 2-Parameter algorithm, generated by MEGA X for the MOTUs defined by the ABGD, ASAP, mPTP, bPTP, and GMYC analyses.

**MOTU**	**Genetic Distance**					
	1	2	3	4	5	6
1. *Leporinus lacustris*+*Leporinus venerei*+*Leporinus piau*+*Leporinus* cf *friderici*	**2.7**					
2. *Leporinus piau*+*Leporinus friderici*	7.91	**2.2**				
3. *Leporinus maculatus*	10.89	10.58	**5.9**			
4. *Leporinus affinis*	12.61	11.26	11.05	**1.8**		
5. *Leporinus unitaeniatus*	13.04	13.49	11.97	11.27	**0.4**	
6. *Leporinus piau*	15.49	16.24	15.64	17.45	14.53	**0.5**

**Table 2:** Meristic traits of the adult *Leporinus* species. Meristic traits of the adult *Leporinus* used to identify the samples analyzed in the present study, following Garavello (1979), Garavello (1989), Garavello & Santos (2007), Britski, Sato & Rosa (1984), and Britski & Birindelli (2008).

**Species**	**Body**	**Coloration**	**Number of scales around the peduncle**	**Dental formula**	**Number of scales in the lateral line**	**Fin coloration**
*L. affinis* Günther (1864)	Pre-dorsal somewhat convex; dorsal inclined slightly between the dorsal and adipose fins, and concave between the adipose and caudal fins.	Body yellowish, with 7 dark transversal stripes on the body and 3–4 on the head.	16	4/4	^*^	Peitoral and pelvic fins light yellow; all other fins hyaline.
*L. friderici* Bloch (1794)	Body tall and robust; large, with Standard Length (SL) of *ca.* 40 cm; body height 26–30% of SL, head length 27–29% of SL; mouth terminal.	Body browny chestnut, with 2–4 dark spots, rounded or oval, on the lateral line.	16	4/4	38 to 40	Anal fin dark gray; all other fins yellowish-gray.
*L. lacustris* Campos (1945)	Body elongated, with a maximum standard length of 20 cm; mouth terminal; incisors truncated.	2–3 dark, rounded, mediolateral spots on the dorsal fin, the first larger and more conspicuous.	16	4/4	33 to 36	All fins yellowish, except the adipose and anal fins, que which are darkened.
*L. maculatus* Müller and Troschel (1844)	Body small, with Standard Length (SL) of *ca.* 10 cm; body height 22–26% of SL, head length 23–25% of SL; mouth subterminal.	Body with 4 black, rounded spots connected by 3 transversal stripes, which cross the lateral line.	16	4/4	39 to 40	^*^
*L. piau*	Body relatively tall.	Body with 3 black spots on the flank, which are elongated horizontally.	16	4/4	35 to 37	^*^
*L. unitaeniatus* Garavello & Santos (2007)	Body elongated and fusiform; small, with maximum standard length of 12 cm; relatively low body (23% of the standard length); mouth subterminal.	Body yellowish, with a conspicuous black longitudinal streak running along the lateral line; 11–13 dark chestnut transversal stripes separated from the lateral line by two rows of scales.	16	4/4	40 to 44	Hyaline.
*L. venerei* Britski & Birindelli (2008)	Body tall; mouth terminal; anal fin long and dark.	3 small, dark spots on the lateral line, of which, the last 2, in particular, the last, are typically faded.	16	4/3	36 to 37	^*^

**Notes.**

* Data not available.

The *L. venerei*, *L. lacustris*, *L.* cf. *friderici*, and *L. piau* MOTU was subdivided into three subclades (*i*, *ii*, *iii* and *iv*—Fig. 3). Subclade *i* includes *L. piau* from Maranhão and *L. venerei* from the Tocantins basin, while subclade *ii* has *L.* cf. *friderici* from the Amazon and *L. piau* from the Turiaçu basins. Subclade *iii* groups *L. lacustris* and *L. piau* from the Jaguaribe basin, and subclade *iv* groups *L. piau* from Maranhão and *L.* cf. *friderici* from the Mearim. The composition of subclade *i* (*L. piau* and *L. venerei*—Fig. 3), together with the diagnostic morphological features of the species, indicates that the specimens from the basins of Maranhão identified as *L. piau* may in fact be *L. venerei*, which would be the first record of this species from this Brazilian state.

One other clade, formed by *L. friderici* from French Guiana and Paraná with *L. piau* from the Mearim, São Francisco, Parnaíba, and Amazon basins, is also well supported (Fig. 3). This raises two important points: (1) the clear polyphyly of *L. friderici* and *L. piau*, which, in the latter case implies a possible error of identification based on the type specimen, and (2) the existence of cryptic diversity in the genus *Leporinus*, in particular in *L. friderici*. Silva-Santos et al. (2018) concluded that the samples identified morphologically as *L. friderici* are in fact a polyphyletic group, given that the specimens collected from the basins of the Brazilian Shield are different from those of *L. friderici* from the type locality. The polyphyly of *L. cf. friderici* was also confirmed in the present study, which is consistent with Silva-Santos et al. (2018), in which a species complex is revealed by the genetic differentiation of the populations present in distinct hydrographic basins. In this case, individuals identified consistently as *L. cf. friderici* may not in fact be conspecific with *L. friderici* from the type locality, that is, they represent different species. Ramirez, Birindelli & Galetti (2017) confirmed the presence of cryptic diversity in this taxon, which may represent a typical scenario of recent diversification, when closely-related taxa may be poorly-distinguished morphologically, creating predictable taxonomic uncertainties, such as those observed in the populations of *L. friderici*.

In the present study, the relationship found among *L. piau*, *L. friderici*, and *L.* cf. *friderici* (Figs. 2–4) alludes to a possible taxonomic inconsistency derived from Fowler’s (1941) description of *L. piau*, as well as the geographic origin of the specimen analyzed in the present study, which was from the São Francisco basin. Fowler (1941) described *Leporinus piau* based on a type specimen from the Salgado River in the Jaguaribe basin of the Brazilian state of Ceará, but included a paratype from the Jatobá River, in the São Francisco basin, which led to the subsequent identification of most *Leporinus* specimens from the São Francisco River as *L. piau* (Garavello & Britski, 2003; Carvalho et al., 2011). However, Silva-Santos et al. (2018), who analyzed nuclear and mitochondrial genetic markers, including COI, noted that the *Leporinus* specimens from the São Francisco basin represent a species distinct from *L. piau* from the type locality in the Jaguaribe basin. Clearly, Fowler’s (1941) inclusion of a paratype from a distinct hydrographic basin have contributed fundamentally to the taxonomic uncertainties surrounding *L. piau*.

In the present study, *L. maculatus*, *L.unitaeniatus*, and *L. affinis* are valid nominal species, which presented considerable congruence between the traditional and molecular taxonomies. These three species constitute distinct MOTUs, which reflect their arrangement in different clades (BI and ML analyses: Figs. 2–4). All these species present easily distinguished diagnostic traits, such as the numerous spots dotting the body of *L. maculatus*, the single longitudinal stripe of *L. unitaeniatus*, and the lateral bands with no subdivisions observed in *L. affinis* (Britski & Garavello, 2005; Sidlauskas & Vari, 2012).

The samples from the Parnaíba basin identified here as *L. piau* and defined as a single MOTU by all the species delimitation models were grouped in a single clade with a genetic distance of 0.5%. These samples were delimited clearly as a more basal species separate from all the others, with evidence that they had been wrongly identified, and are in fact representatives of the genus *Megaleporinus*. This genus was described recently by Ramirez, Birindelli & Galetti (2017), based on a combined morphological, chromosomal, and molecular approach, which assigned the large-bodied *Leporinus* to a monophyletic clade, which was denominated *Megaleporinus*. In the present study, these samples were delimited clearly as a single, basal species well separated from all the others, although a more detailed analysis would be necessary to better determine their taxonomic status.

The samples from the basins of Maranhão and Piauí, together with those from the Tocantins River collected for the present study revealed the cryptic diversity found in *Leporinus*, given that the specimens from the basins of the Itapecuru, Mearim, Pericumã, Turiaçu, Periá, and Preguiças rivers in Maranhão, and the Parnaíba River in Piauí were identified as *L. piau* based on their morphological traits. The study of the DNA barcode and the analytical tools employed here confirmed that *L. friderici* likely constitutes a polyphyletic species complex, leading to the frequent misidentification of specimens as *L. piau*. It will only be possible to resolve this scenario definitively with a systematic re-evaluation of the specimens collected from the hydrographic basins of the states of Maranhão and Piauí.

In the specific case of subclade *i* (Fig. 3; Tables S3-S4), which groups *L. piau* from Maranhão with *L. venerei* from the Tocantins basin, the most parsimonious interpretation of the results of this analysis, together with the diagnostic traits of the two species, would be to consider them to be a single taxon, that is, *L. venerei*. This would thus be the first record of *L. venerei* from the basins of the Itapecuru, Mearim, Turiaçu, and Periá rivers, in the state of Maranhão.

Prior to the present study, three *Leporinus* species were considered to be present in the hydrographic basins of the Brazilian state of Maranhão—*L. affinis*, in the Itapecuru basin(Abreu et al., 2019), *L. friderici* in the Itapecuru, Mearim, Maracaçumé, Munim, Periá, and Parnaíba basins (Piorski et al., 1998; Soares, 2005; Ramos, Ramos & Ramos, 2014; Melo et al., 2016; Abreu et al., 2019; Brito et al., 2019; Brito et al., 2020; Guimarães et al., 2021a; Guimarães et al., 2021b; Guimarães et al., 2021c), and *L. piau* in the basins of the Itapecuru, Mearim, Turiaçu, and Parnaíba rivers (Barros, Fraga & Birindelli, 2011; Ramos, Ramos & Ramos, 2014; Ribeiro et al., 2014; Assega & Birindelli, 2019; Abreu et al., 2019). Based on analyses of molecular data, however, Fraga et al. (2014) and Nascimento et al. (2016) found evidence of two distinct lineages in the *L. piau* group from the Itapecuru basin, while in the present study, *L. piau* was assigned to three different clades, being associated strongly with *L.* cf. *friderici* in two clades and with *L. venerei* in one. This leads us to conclude that *L. piau* is, in fact, absent from the basins of Maranhão, which are instead populated by *L.* cf. *friderici* and *L. venerei*, with the latter being recorded in Maranhão for the first time. This restricts *L. piau* to the basin of the Parnaíba River.

## Conclusions

The molecular analyses presented here, including the different species delimitation approaches, identified the presence of four *Leporinus* species in the hydrographic basins of central northern Brazil—*L. maculatus, L. unitaenitus, L. affinis*, and *L. venerei*. However, the species delimitation analyses also assigned *L.* cf. *friderici* and *L. piau* to two different molecular operational units, which leads us to believe that an additional species, morphologically indistinguishable from *L.* cf. *friderici*, may be present. The analyses also revealed a distinct group of two of the specimens, which indicates emphatically the presence of *L. piau* in the basin of the Parnaíba River, which indicates the presence of a total of six nominal species in the hydrographic basins of central northern Brazil. The confirmation of the presence of *L. venerei* in the Itapecuru, Mearim, Turiaçu, Preguiças and Periá basins represents a new record for the state of Maranhão, amplifying the known distribution of this species in Brazil.

##  Supplemental Information

10.7717/peerj.15184/supp-1 Table S1 Localities sampled of the hydrographic basins of central northern Brazil. Localities sampled in the present study of *Leporinus* reservoirs in hydrographics basins in the state of Maranhao, Piaui and Tocantins.

10.7717/peerj.15184/supp-2 Table S2 Localities sampled in the 25 sequences were obtained from GenBank

10.7717/peerj.15184/supp-3 Table S3 Genetic distance matrix Genetic distance, based on the Kimura 2-Parameter algorithm, for the genus Leporinus and Megaleporinus in the hydrographic basins of central northern Brazil.

10.7717/peerj.15184/supp-4 Table S4 Arrangement of the specimens in groups and the number of Molecular Operational Units proposed for *Leporinus.* ML, Maximum Likelihood. Unnamed, group with more than one valid species.

10.7717/peerj.15184/supp-5 Supplemental Information 5 Sequence Data The sequence data is also available at GenBank: OP781850 to OP781884, OP782222 to OP782283, OP782350 to OP7882375 and OP782385 to OP782418.

## Additional Information and Declarations

### Data Availability

The following information was supplied regarding data availability: The data is available at NCBI GenBank: OP781850 to OP781884, OP782222 to OP782283, OP782350 to OP7882375 and OP782385 to OP782418.

## References

[ref-1] Abreu JMS, Craig JM, Albert JS, Piorski NM (2019). Historical biogeography of fishes from coastal basins of Maranhão State, northeastern Brazil. Neotropical Ichthyology.

[ref-2] Ardura A, Linde AR, Moreira JC, Garcia-Vazquez E (2010). DNA barcoding for conservation and management of Amazonian commercial fish. Biological Conservation.

[ref-3] Assega FY, Birindelli JLO (2019). Taxonomic revision of the genus Anostomoides (Characiformes: Anostomidae). Zootaxa.

[ref-4] Barros MC, Fraga EC, Birindelli JLO (2011). Fishes from the Itapecuru River basin, State of Maranhão, Northeastern Brazil. Brazilian Journal of Biology.

[ref-5] Bickford D, Lohman DJ, Sodhi NS, Ng PKL, Meier R, Winker K, Ingram KK, Das I (2007). Cryptic species as a window on diversity and conservation. Trends in Ecology and Evolution.

[ref-6] Birindelli JLO, Britski HA, Ramirez JL (2020). A new endangered species of Megaleporinus (Characiformes: Anostomidae) from the Rio de Contas basin, eastern Brazil. Journal of Fish Biology.

[ref-7] Borodin NA (1929). Notes on some species and subspecies of the genus *Leporinus* Spix. Memoirs of the Museum of Comparative Zoölogy.

[ref-8] Brito OS, Guimarães EC, Ferreira BRA, Ottoni FP, Piorski NM (2019). Freshwater fishes of the Parque Nacional dos Lençóis Maranhenses and adjacent areas. Biota Neotropica.

[ref-9] Brito OS, Guimarães EC, Ferreira BRA, Santo SJP, Amaral YT, Ottoni FP (2020). Updated and supplementary data on Brito et al. (2019): Freshwater fishes of the PN dos Lençóis Maranhenses and adjacent areas. Ichthyological Contributions of Pecescriollos.

[ref-10] Britski HA, Birindelli JLO (2008). Description of a new species of the genus Leporinus Spix (Characiformes: Anostomidae) from the rio Araguaia, Brazil, with comments on the taxonomy and distribution of L. parae and L. lacustris. Neotropical Ichthyology.

[ref-11] Britski HA, Birindelli JL (2019). Description of a new species of *Leporinus* (Characiformes: Anostomidae) from the Rio Tapajós basin, Brazil. Zootaxa.

[ref-12] Britski HA, Garavello JC (1978). Sobre *Leporinus octofasciatus* steindachner da bacia do Paraná (Pisces, Anostomidae). Papéis Avulsos de Zoologia.

[ref-13] Britski HA, Garavello JC (2005). Uma nova espécie de Leporinus Agassiz, 1829, da Bacia Amazônica (Ostariophysi: Characiformes: Anostomidae). Comunicações do Museu de ciências e Tecnologia da PUCRS: Série Zoologia.

[ref-14] Britski HA, Sato Y, Rosa ABS (1984). Manual de identificação de peixes da Região de Três Marias.

[ref-15] Burns MD, Chatfield M, Birindelli JLO, Sidlauskas BL (2017). Systematic assessment of the Leporinus desmotes species complex, with a description of two new species. Neotropical Ichthyology.

[ref-16] Carvalho APC, Collins RA, Martínez JG, Farias IP, Hrbek T (2018). From shallow to deep divergences: mixed messages from Amazon Basin cichlids. Hydrobiologia.

[ref-17] Carvalho DC, Oliveira DA, Pompeu PS, Leal CG, Oliveira C, Hanne R (2011). Deep barcode divergence in Brazilian freshwater fishes: the case of the Sao Francisco River basin. Mitochondrial DNA.

[ref-18] Darriba D, Taboada GL, Doallo R, Posada D (2012). JModelTest2: more models, new heuristics and parallel computing. Nature Methods.

[ref-19] Dinno A (2009). Exploring the sensitivity of horn’s parallel analysis to the distributional form of simulated data. Multivariate Behavioral Research.

[ref-20] Drummond AJ, Simon YW, Phillips MJ, Rambaut A (2006). Relaxed phylogenetics and dating with confidence. PLOS Biology.

[ref-21] Drummond AJ, Suchard AM, Xie D, Rambaut A (2012). Bayesian phylogenetics with BEAUti and the BEAST 1.7. Molecular Biology and Evolution.

[ref-22] Ezard T, Fujisawa T, Barraclough TG (2009). SPLITS: SPecies’ LImits by Threshold Statistics. R package version 1.0-18/r45. http://R-Forge.R-project.org/projects/splits/.

[ref-23] Fowler HW (1941). Academy of natural sciences a collection of fresh-water fishes obtained in Eastern Brazil by Dr. Rodolpho Von Ihering. Proceedings of the Academy of Natural Sciences of Philadelphia.

[ref-24] Fraga E, Silva LMM, Schneider H, Sampaio I, Barros MC (2014). Variabilidade genética em populações naturais de Leporinus piau (Anostomidae, Characiformes) da bacia do Rio Itapecuru. Revista Trópica.

[ref-25] Frantine-Silva W, Sofia SH, Orsi ML, Almeida FS (2015). DNA barcoding of freshwater ichthyoplankton in the Neotropics as a tool for ecological monitoring. Molecular Ecology Resources.

[ref-26] Fricke R, Eschmeyer W, Laan RVanDer (2021). CAS - Eschmeyer’s Catalog of Fishes - Species by Family. 2021. https://researcharchive.calacademy.org/research/ichthyology/catalog/fishcatmain.asp.

[ref-27] Fujisawa T, Barraclough TG (2013). Delimiting species using single-locus data and the generalized mixed yule coalescent (GMYC) approach: a revised method and evaluation on simulated datasets. Systematic Biology.

[ref-28] Galetti Jr PM, Lima NRW, Venere PC (1995). A monophyletic ZW sex chromosome system in *Leporinus* (Anostomidae, Characiformes). Cytologia.

[ref-29] Garavello JC (1979). Revisão taxonômica do gênero *Leporinus* Spix, 1829 (ostariophysi, Anostomidae) Tese de Doutorado.

[ref-30] Garavello JC (1989). Leporinus microphthalmus sp.n. Bacia do Rio Paranaiba, Alto Paraná (Pisces, Anostomidae). Revista Brasileira de Biologia.

[ref-31] Garavello JC, Britski HA (2003). Family Anostomidae. REIS and others, RE. Checklist of the Freshwater Fishes of South and Central America. Porto Alegre, Editora da Pontifícia Universidade Católica do rio Grande do Sul.

[ref-32] Garavello JC, Santos GM (2007). Two news species of Leporinus Agassiz, 1829 from Araguaia-Tocantins system, Amazon basin, Brazil (Ostariophysi, Anostomidae). Brazilian Journal of Biology.

[ref-33] Gernhard T (2008). The conditioned reconstruted process. Journal of Theoretical Biology.

[ref-34] Géry J (1977). Characoids of the world.

[ref-35] Guimarães EC, Brito OS, Oliveira RF, Aguiar RG, Ottoni FP, Guimarães KLA, Santos JP, Rodrigues LRR (2021a). Peixes do rio Pindaré e suas potencialidades ornamentais.

[ref-36] Guimarães EC, Brito OS, Santos JP, Oliveira FR, FP Ottoni (2021c). Supplementary material to Guimarães others (2020): Peixes, Fauna de vertebrados ao longo da Estrada de Ferro Carajás. Ichthyological Contributions of PecesCriollos.

[ref-37] Guimarães EC, Oliveira RF, Brito OS, Vieira LO, Santos JP, Oliveira ES, Aguiar RG, Katz AM, Lopes DF, Nunes JLS, Ottoni FP (2021b). Biodiversidade, potencialidades ornamentais e guia ilustrado dos peixes da Mata Itamacaoca município de Chapadinha-MA.

[ref-38] Hall TA (1999). BioEdit: a user-friendly biological sequence alignment editor and analysis program for Windows 95/98/NT. Nucleic Acids Symposium Series.

[ref-39] Hebert PDN, Cywinska A, Ball SL, De Waard JR (2003). Biological 58 identifications through DNA barcodes. Proceedings of the Royal Society B: Biological Sciences.

[ref-40] Kress WJ, García-Robledo C, Uriarte M, Erickson DL (2015). DNA barcodes for ecology, evolution, and conservation. Trends in Ecology and Evolution.

[ref-41] Kumar S, Stecher G, Li M, Knyar C, Tamura K (2018). MEGA X: molecular evolutionary genetics analysis across computing platforms. Molecular Biology and Evolution.

[ref-42] Librado P, Rozas J (2009). DNAsp v5: a software for comprehensive analyses of DNA polymorphism data. Bioinformatics.

[ref-43] Melo FAG, Buckup PA, Ramos TPA, Souza AKN, Silva CMA, Costa TC, Torres AR (2016). Fish fauna of the lower course of the Parnaíba river, northeastern Brazil. Boletim do Museu de Biologia.

[ref-44] Miller MA, Pfeiffer W, Schwartz T (2010). Creating the CIPRES Science Gateway for inference of large phylogenetic trees.

[ref-45] Myers GS (1950). Studies on South American fresh-water fishes. II. The genera of Anostominae Characids. Stanford Ichthyological Bulletin.

[ref-46] Nascimento MHS, Almeida MS, Veira MNS, Limeira Filho D, Lima RC, Barros MC, Fraga EC (2016). DNA barcoding reveals high levels of genetic diversity in the fishes of the Itapecuru Basin in Maranhão, Brazil. Genetics and Molecular Research.

[ref-47] Papa Y, Bail PYLe, Covain R (2021). Genetic landscape clustering of a large DNA barcoding data set reveals shared patterns of genetic divergence among freshwater fishes of the Maroni Basin. Molecular Ecology Resources.

[ref-48] Paradis E, Schliep K (2019). Ape 5.0: an environment for modern phylogenetics and evolutionary analyses in R. Bioinformatics.

[ref-49] Pereira LH, Hanner R, Foresti F, Oliveira C (2013). Can DNA barcoding accurately discriminate megadiverse Neotropical freshwater fish fauna?. BMC Genomic Data.

[ref-50] Piorski NM, Castro ACL, Pereira LG, Muniz MEL (1998). Ictiofauna do trecho inferior do Rio Itapecuru, nordeste do Brasil. Boletim do Laboratório de Hidrobiologia.

[ref-51] Puillandre N, Brouillet S, Achaz G (2020). ASAP: Assemble Species by Automatic Partitioning. Molecular Ecology Resources.

[ref-52] Puillandre N, Lambert A, Brouillet S, Achaz G (2012). ABGD, Automatic barcode gap discovery for primary species delimitation. Molecular Ecology.

[ref-53] R Core Team (2021). https://www.r-project.org.

[ref-54] Rambaut A (2014). http://tree.bio.ed.ac.uk/software/figtree/.

[ref-55] Rambaut A, Suchard MA, Xie D, Drummond AJ (2014). http://tree.bio.ed.ac.uk/software/tracer.

[ref-56] Ramirez JL, Birindelli JLO, Galetti PM (2017). Um novo gênero de Anostomidae (Ostariophysi: Characiformes): diversidade, filogenia e biogeografia com base em dados citogenéticos, moleculares e morfológicos. Molecular Phylogenetic Evolution.

[ref-57] Ramirez JL, Carvalho-Costa LF, Venere PC, Carvalho DC, Troy WP, Galetti PM (2016). Testing monophyly of the freshwater fish Leporinus (Characiformes, Anostomidae) through molecular analysis. Journal of Fish Biology.

[ref-58] Ramirez JL, Santos CA, Machado CB, Oliveira AK, Garavello JC, Britski HA, Galetti PM (2020). Molecular phylogeny and species delimitation of the genus *Schizodon* (Characiformes, Anostomidae). Molecular Phylogenetics and Evolution.

[ref-59] Ramos TPA, Ramos RTC, Ramos SAQA (2014). Ichthyofauna of the Parnaíba river basin, northeastern Brazil. Biota Neotropical.

[ref-60] Ribeiro MFR, Piorski NM, Almeida ZS, Nunes JLS (2014). Fish aggregating known as moita, an artesanal fishing technique performed in the Munim River, State of Maranhão, Brazil. Boletim Instituto de Pesca.

[ref-61] Sanger F, Nicklen S, Coulson AR (1977). DNA sequencing with chain-terminating inhibitors. Proceedings of the National Academy of Sciences of the United States of America.

[ref-62] Sidlauskas BL, Vari RP (2008). Phylogenetic relationships within the South American fish family Anostomidae (Teleostei, Ostariophysi, Characiformes). Zoological Journal of the Linnean Society.

[ref-63] Sidlauskas BL, Vari RP (2012). Diversity and distribution of anostomoid fishes (Teleostei: Characiformes) throughout the Guianas. Cybium.

[ref-64] Silva-Santos R, Ramirez JL, Freitas PD, Galetti Jr PM, Freitas PD (2018). Molecular evidences of a hidden complex scenario in *Leporinus cf. friderici*. Frontiers in Genetics.

[ref-65] Soares EC (2005). Peixes do Mearim.

[ref-66] Stamatakis A (2014). RAxML Version 8: a tool for phylogenetic analysis and postanalysis of large phylogenies. Bioinformatics.

[ref-67] Suchard MA, Lemey P, Baele G, Ayres DL, Drummond AJ, Rambaut A (2018). Bayesian phylogenetic and phylodynamic data integration using BEAST 1.10. Virus Evolution.

[ref-68] Thompson JD, Higgins DG, Gibson TJ (1994). CLUSTAL W: improving the sensitivity of progressive multiple sequence alignment through sequence weighting, position-specific gap penalties and weight matrix choice. Nucleic Acids Research.

[ref-69] Venables WN, Ripley BD (2002). Modern Applied Statistics with S.

[ref-70] Ward RD, Zemlak TS, Innes BH, Last PR, Hebert PDN (2005). DNA barcoding Australia’s fish species. Philosophical Transactions of the Royal Society of London, Series B, Biological Sciencies.

[ref-71] Zhang J, Kapli P, Pavlidis P, Stamatakis A (2013). A general species delimitation method with applications to phylogenetic placements. Bioinformatics.

