# Supplementary material for: The DNA barcode reveals cryptic diversity and a new record for the genus Leporinus (Characiformes, Anostomidae) in the hydrographic basins of central northern Brazil
Source: PeerJ. 2023 May 25;11:e15184. doi: 10.7717/peerj.15184 (PMC10225125; doi:10.7717/peerj.15184)
Supplement: Table S1 — Localities sampled in the present study of Leporinus reservoirs in hydrographics basins in the state of Maranhao, Piaui and Tocantins. [file peerj-11-15184-s001.docx]

| **GENBANK** | **CODE** | **SPECIE** | **HYDROGRAPHICS BASINS** | **GEOGRAPHIC**  **COORDINATE** |
| --- | --- | --- | --- | --- |
| OP781850 | LEP01 | *Leporinus piau* | Itapecuru | 03º31'39"S 44º24'19"W |
| OP781851 | LEP02 | *Leporinus piau* | Itapecuru | 03º31'39"S 44º24'19"W |
| OP781852 | LEP03 | *Leporinus piau* | Itapecuru | 03º31'39"S 44º24'19"W |
| OP781853 | LEP05 | *Leporinus piau* | Itapecuru | 03º31'39"S 44º24'19"W |
| OP781854 | LEP06 | *Leporinus piau* | Itapecuru | 03º31'39"S 44º24'19"W |
| OP781855 | LEP07 | *Leporinus piau* | Itapecuru | 03º31'39"S 44º24'19"W |
| OP781856 | LEP08 | *Leporinus piau* | Itapecuru | 03º31'39"S 44º24'19"W |
| OP781857 | LEP11 | *Leporinus piau* | Itapecuru | 03º31'39"S 44º24'19"W |
| OP781858 | LEP12 | *Leporinus piau* | Itapecuru | 03º31'39"S 44º24'19"W |
| OP781859 | LEP15 | *Leporinus piau* | Itapecuru | 03º31'39"S 44º24'19"W |
| OP781860 | LEP16 | *Leporinus piau* | Itapecuru | 03º31'39"S 44º24'19"W |
| OP781861 | LEP17 | *Leporinus piau* | Itapecuru | 03º31'39"S 44º24'19"W |
| OP781862 | LEP18 | *Leporinus piau* | Itapecuru | 03º31'39"S 44º24'19"W |
| OP781863 | LEP19 | *Leporinus piau* | Itapecuru | 03º31'39"S 44º24'19"W |
| OP781864 | LEP20 | *Leporinus piau* | Itapecuru | 03º31'39"S 44º24'19"W |
| OP781865 | LEP21 | *Leporinus piau* | Itapecuru | 03º31'39"S 44º24'19"W |
| OP781866 | LEP23 | *Leporinus piau* | Itapecuru | 03º31'39"S 44º24'19"W |
| OP781867 | LEP25 | *Leporinus piau* | Itapecuru | 03º31'39"S 44º24'19"W |
| OP781868 | LEP29 | *Leporinus piau* | Itapecuru | 03º31'39"S 44º24'19"W |
| OP781869 | LEP28 | *Leporinus piau* | Itapecuru | 03º31'39"S 44º24'19"W |
| OP781870 | LEP30 | *Leporinus piau* | Itapecuru | 03º31'39"S 44º24'19"W |
| OP781871 | LEP45 | *Leporinus piau* | Itapecuru | 03º31'39"S 44º24'19"W |
| OP781872 | LEP46 | *Leporinus piau* | Itapecuru | 03º31'39"S 44º24'19"W |
| OP781873 | LEP47 | *Leporinus piau* | Itapecuru | 03º31'39"S 44º24'19"W |
| OP781874 | LEP48 | *Leporinus piau* | Itapecuru | 03º31'39"S 44º24'19"W |
| OP781875 | LEP49 | *Leporinus piau* | Itapecuru | 03º31'39"S 44º24'19"W |
| OP781876 | LEP50 | *Leporinus piau* | Itapecuru | 03º31'39"S 44º24'19"W |
| OP781877 | LEP51 | *Leporinus piau* | Itapecuru | 03º31'39"S 44º24'19"W |
| OP781878 | LEP53 | *Leporinus piau* | Itapecuru | 03º31'39"S 44º24'19"W |
| OP781879 | LEP78 | *Leporinus piau* | Itapecuru | 03º31'39"S 44º24'19"W |
| OP781880 | LEP62 | *Leporinus piau* | Itapecuru | 03º31'39"S 44º24'19"W |
| OP781881 | LEP67 | *Leporinus piau* | Itapecuru | 03º31'39"S 44º24'19"W |
| OP781882 | LEP68 | *Leporinus piau* | Itapecuru | 03º31'39"S 44º24'19"W |
| OP781883 | LEP79 | *Leporinus piau* | Itapecuru | 03º31'39"S 44º24'19"W |
| OP781884 | LEP80 | *Leporinus piau* | Itapecuru | 03º31'39"S 44º24'19"W |
| OP782222 | LEP101 | *Leporinus piau* | Mearim | 04º34'08"S 44º35'31"W |
| OP782223 | LEP143 | *Leporinus piau* | Mearim | 04º34'08"S 44º35'31"W |
| OP782224 | LEP144 | *Leporinus piau* | Mearim | 04º34'08"S 44º35'31"W |
| OP782225 | LEP145 | *Leporinus piau* | Mearim | 04º34'08"S 44º35'31"W |
| OP782226 | LEP147 | *Leporinus piau* | Mearim | 04º34'08"S 44º35'31"W |
| OP782227 | LEP148 | *Leporinus piau* | Mearim | 04º34'08"S 44º35'31"W |
| OP782228 | MEA55 | *Leporinus piau* | Mearim | 04º34'08"S 44º35'31"W |
| OP782229 | MEA56 | *Leporinus piau* | Mearim | 04º34'08"S 44º35'31"W |
| OP782230 | MEA57 | *Leporinus piau* | Mearim | 04º34'08"S 44º35'31"W |
| OP782231 | MEA58 | *Leporinus piau* | Mearim | 04º34'08"S 44º35'31"W |
| OP782232 | MEA59 | *Leporinus piau* | Mearim | 04º34'08"S 44º35'31"W |
| OP782233 | MEA60 | *Leporinus piau* | Mearim | 04º34'08"S 44º35'31"W |
| OP782234 | MEA61 | *Leporinus piau* | Mearim | 04º34'08"S 44º35'31"W |
| OP782235 | MEA63 | *Leporinus piau* | Mearim | 04º34'08"S 44º35'31"W |
| OP782236 | MEA64 | *Leporinus piau* | Mearim | 04º34'08"S 44º35'31"W |
| OP782237 | MEA65 | *Leporinus piau* | Mearim | 04º34'08"S 44º35'31"W |
| OP782238 | MEA66 | *Leporinus piau* | Mearim | 04º34'08"S 44º35'31"W |
| OP782239 | MEA99 | *Leporinus piau* | Mearim | 04º34'08"S 44º35'31"W |
| OP782240 | MEA100 | *Leporinus piau* | Mearim | 04º34'08"S 44º35'31"W |
| OP782241 | MEA349 | *Leporinus piau* | Mearim | 04º34'08"S 44º35'31"W |
| OP782242 | MEA350 | *Leporinus piau* | Mearim | 04º34'08"S 44º35'31"W |
| OP782243 | MEA351 | *Leporinus piau* | Mearim | 04º34'08"S 44º35'31"W |
| OP782244 | MEA352 | *Leporinus piau* | Mearim | 04º34'08"S 44º35'31"W |
| OP782245 | MEA353 | *Leporinus piau* | Mearim | 04º34'08"S 44º35'31"W |
| OP782246 | MEA492 | *Leporinus piau* | Mearim | 04º34'08"S 44º35'31"W |
| OP782247 | MEA493 | *Leporinus piau* | Mearim | 04º34'08"S 44º35'31"W |
| OP782248 | MAE495 | *Leporinus piau* | Mearim | 04º34'08"S 44º35'31"W |
| OP782249 | MEA494 | *Leporinus piau* | Mearim | 04º34'08"S 44º35'31"W |
| OP782250 | MEA496 | *Leporinus piau* | Mearim | 04º34'08"S 44º35'31"W |
| OP782251 | MEA497 | *Leporinus piau* | Mearim | 04º34'08"S 44º35'31"W |
| OP782252 | LEP98 | *Leporinus piau* | Mearim | 03º39'54''S 45º25'31''W |
| OP782253 | LEP112 | *Leporinus piau* | Mearim | 03º39'54''S 45º25'31''W |
| OP782254 | LEP113 | *Leporinus piau* | Mearim | 03º39'54''S 45º25'31''W |
| OP782255 | LEP114 | *Leporinus piau* | Mearim | 03º39'54''S 45º25'31''W |
| OP782256 | LEP116 | *Leporinus piau* | Mearim | 03º39'54''S 45º25'31''W |
| OP782257 | LEP115 | *Leporinus piau* | Mearim | 03º39'54''S 45º25'31''W |
| OP782258 | LEP119 | *Leporinus piau* | Mearim | 03º39'54''S 45º25'31''W |
| OP782259 | LEP120 | *Leporinus piau* | Mearim | 03º39'54''S 45º25'31''W |
| OP782260 | LEP121 | *Leporinus piau* | Mearim | 03º39'54''S 45º25'31''W |
| OP782261 | LEP122 | *Leporinus piau* | Mearim | 03º39'54''S 45º25'31''W |
| OP782262 | LEP123 | *Leporinus piau* | Mearim | 03º39'54''S 45º25'31''W |
| OP782263 | LEP124 | *Leporinus piau* | Mearim | 03º39'54''S 45º25'31''W |
| OP782264 | LEP125 | *Leporinus piau* | Mearim | 03º39'54''S 45º25'31''W |
| OP782265 | LEP126 | *Leporinus piau* | Mearim | 03º39'54''S 45º25'31''W |
| OP782266 | LEP127 | *Leporinus piau* | Mearim | 03º39'54''S 45º25'31''W |
| OP782267 | LEP129 | *Leporinus piau* | Mearim | 03º39'54''S 45º25'31''W |
| OP782268 | LEP130 | *Leporinus piau* | Mearim | 03º39'54''S 45º25'31''W |
| OP782269 | LEP131 | *Leporinus piau* | Mearim | 03º39'54''S 45º25'31''W |
| OP782270 | LEP132 | *Leporinus piau* | Mearim | 03º39'54''S 45º25'31''W |
| OP782271 | LEP133 | *Leporinus piau* | Mearim | 03º39'54''S 45º25'31''W |
| OP782272 | LEP134 | *Leporinus piau* | Mearim | 03º39'54''S 45º25'31''W |
| OP782273 | LEP136 | *Leporinus piau* | Mearim | 03º39'54''S 45º25'31''W |
| OP782274 | LEP137 | *Leporinus piau* | Mearim | 03º39'54''S 45º25'31''W |
| OP782275 | LEP138 | *Leporinus piau* | Mearim | 03º39'54''S 45º25'31''W |
| OP782276 | LEP139 | *Leporinus piau* | Mearim | 03º39'54''S 45º25'31''W |
| OP782277 | LEP141 | *Leporinus piau* | Mearim | 03º39'54''S 45º25'31''W |
| OP782278 | LEP142 | *Leporinus piau* | Mearim | 03º39'54''S 45º25'31''W |
| OP782279 | COR01 | *Leporinus piau* | Mearim | 03º39'54''S 45º25'31''W |
| OP782280 | COR02 | *Leporinus piau* | Mearim | 03º39'54''S 45º25'31''W |
| OP782281 | COR03 | *Leporinus piau* | Mearim | 03º39'54''S 45º25'31''W |
| OP782282 | COR04 | *Leporinus piau* | Mearim | 03º39'54''S 45º25'31''W |
| OP782283 | COR05 | *Leporinus piau* | Mearim | 03º39'54''S 45º25'31''W |
| OP782350 | TUR34 | *Leporinus piau* | Turiacu | 02°15’87’’ S 45°19’37’’ W |
| OP782351 | TUR35 | *Leporinus piau* | Turiacu | 02°15’87’’ S 45°19’37’’ W |
| OP782352 | TUR36 | *Leporinus piau* | Turiacu | 02°15’87’’ S 45°19’37’’ W |
| OP782353 | TUR37 | *Leporinus piau* | Turiacu | 02°15’87’’ S 45°19’37’’ W |
| OP782354 | TUR38 | *Leporinus piau* | Turiacu | 02°15’87’’ S 45°19’37’’ W |
| OP782355 | TUR46 | *Leporinus piau* | Turiacu | 02°15’87’’ S 45°19’37’’ W |
| OP782356 | TUR256 | *Leporinus piau* | Turiacu | 02°15’87’’ S 45°19’37’’ W |
| OP782357 | TUR257 | *Leporinus piau* | Turiacu | 02°15’87’’ S 45°19’37’’ W |
| OP782358 | TUR258 | *Leporinus piau* | Turiacu | 02°15’87’’ S 45°19’37’’ W |
| OP782359 | TUR262 | *Leporinus piau* | Turiacu | 02°15’87’’ S 45°19’37’’ W |
| OP782360 | TUR263 | *Leporinus piau* | Turiacu | 02°15’87’’ S 45°19’37’’ W |
| OP782361 | TUR264 | *Leporinus piau* | Turiacu | 02°15’87’’ S 45°19’37’’ W |
| OP782362 | PER70 | *Leporinus piau* | Pericuma | 02º 31’ 17” S 45°04’57’’ W |
| OP782363 | PER71 | *Leporinus piau* | Pericuma | 02º 31’ 17” S 45°04’57’’ W |
| OP782364 | PER66 | *Leporinus piau* | Pericuma | 02º 31’ 17” S 45°04’57’’ W |
| OP782365 | PER67 | *Leporinus piau* | Pericuma | 02º 31’ 17” S 45°04’57’’ W |
| OP782366 | PER68 | *Leporinus piau* | Pericuma | 02º 31’ 17” S 45°04’57’’ W |
| OP782367 | PER69 | *Leporinus piau* | Pericuma | 02º 31’ 17” S 45°04’57’’ W |
| OP782368 | PER160 | *Leporinus piau* | Pericuma | 02º 31’ 17” S 45°04’57’’ W |
| OP782369 | PER161 | *Leporinus piau* | Pericuma | 02º 31’ 17” S 45°04’57’’ W |
| OP782370 | PER162 | *Leporinus piau* | Pericuma | 02º 31’ 17” S 45°04’57’’ W |
| OP782371 | PER163 | *Leporinus piau* | Pericuma | 02º 31’ 17” S 45°04’57’’ W |
| OP782372 | PER164 | *Leporinus piau* | Pericuma | 02º 31’ 17” S 45°04’57’’ W |
| OP782373 | PERI01 | *Leporinus piau* | Peria | 2°35’24” S43°27’36” W |
| OP782374 | PERI02 | *Leporinus piau* | Peria | 2°35’24” S43°27’36” W |
| OP782375 | PRE01 | *Leporinus piau* | Preguiças | 2°29' 16'' S 43° 17' 34'' W |
| OP782401 | PALEP01 | *Leporinus piau* | Parnaiba | 5°05′21″ S 42°48′06″W |
| OP782402 | PALEP03 | *Leporinus piau* | Parnaiba | 5°05′21″ S 42°48′06″W |
| OP782403 | PALEP04 | *Leporinus piau* | Parnaiba | 5°05′21″ S 42°48′06″W |
| OP782404 | PALEP05 | *Leporinus piau* | Parnaiba | 5°05′21″ S 42°48′06″W |
| OP782405 | PALEP06 | *Leporinus piau* | Parnaiba | 5°05′21″ S 42°48′06″W |
| OP782406 | PALEP08 | *Leporinus piau* | Parnaiba | 5°05′21″ S 42°48′06″W |
| OP782407 | PALEP09 | *Leporinus piau* | Parnaiba | 5°05′21″ S 42°48′06″W |
| OP782408 | PALEP13 | *Leporinus piau* | Parnaiba | 5°05′21″ S 42°48′06″W |
| OP782409 | PALEP14 | *Leporinus piau* | Parnaiba | 5°05′21″ S 42°48′06″W |
| OP782410 | PALEP15 | *Leporinus piau* | Parnaiba | 5°05′21″ S 42°48′06″W |
| OP782411 | PALEP16 | *Leporinus piau* | Parnaiba | 5°05′21″ S 42°48′06″W |
| OP782412 | PALEP18 | *Leporinus piau* | Parnaiba | 5°05′21″ S 42°48′06″W |
| OP782413 | PALEP19 | *Leporinus piau* | Parnaiba | 5°05′21″ S 42°48′06″W |
| OP782414 | PALEP20 | *Leporinus piau* | Parnaiba | 5°05′21″ S 42°48′06″W |
| OP782415 | PALEP21 | *Leporinus piau* | Parnaiba | 5°05′21″ S 42°48′06″W |
| OP782416 | PALEP22 | *Leporinus piau* | Parnaiba | 5°05′21″ S 42°48′06″W |
| OP782417 | PALEP23 | *Leporinus piau* | Parnaiba | 5°05′21″ S 42°48′06″W |
| OP782418 | PALEP25 | *Leporinus piau* | Parnaiba | 5°05′21″ S 42°48′06″W |
| OP782386 | TO493 | *Leporinus venerei* | Tocantins | 10°7'30"S 48°21'33"W |
| OP782385 | TO495 | *Leporinus venerei* | Tocantins | 10°7'30"S 48°21'33"W |
| OP782387 | TO496 | *Leporinus venerei* | Tocantins | 10°7'30"S 48°21'33"W |
| OP782388 | TO548 | *Leporinus venerei* | Tocantins | 5°27'58"S 47°34'28"W |
| OP782389 | TO557 | *Leporinus maculatus* | Tocantins | 5°27'58"S 47°34'28"W |
| OP782390 | TO640 | *Leporinus affinis* | Tocantins | 7°4'47"S 47°37'17"W |
| OP782391 | TO661 | *Leporinus affinis* | Tocantins | 7°4'47"S 47°37'17"W |
| OP782392 | TO603 | *Leporinus affinis* | Tocantins | 7°4'47"S 47°37'17"W |
| OP782393 | TO634 | *Leporinus affinis* | Tocantins | 7°4'47"S 47°37'17"W |
| OP782394 | TO636 | *Leporinus affinis* | Tocantins | 7°4'47"S 47°37'17"W |
| OP782395 | TO637 | *Leporinus affinis* | Tocantins | 7°4'47"S 47°37'17"W |
| OP782396 | TO638 | *Leporinus unitaeniatus* | Tocantins | 5°27'58.3"S 47°34'28.4"W |
| OP782397 | TO599 | *Leporinus unitaeniatus* | Tocantins | 5°27'58.3"S 47°34'28.4"W |
| OP782398 | TO600 | *Leporinus unitaeniatus* | Tocantins | 5°27'58.3"S 47°34'28.4"W |
| OP782399 | TO601 | *Leporinus unitaeniatus* | Tocantins | 5°27'58.3"S 47°34'28.4"W |
| OP782400 | TO602 | *Leporinus unitaeniatus* | Tocantins | 5°27'58.3"S 47°34'28.4"W |
