# Supplementary material for: The DNA barcode reveals cryptic diversity and a new record for the genus Leporinus (Characiformes, Anostomidae) in the hydrographic basins of central northern Brazil
Source: PeerJ. 2023 May 25;11:e15184. doi: 10.7717/peerj.15184 (PMC10225125; doi:10.7717/peerj.15184)
Supplement: Table S2 [file peerj-11-15184-s002.docx]

| **GENBANK** | **SPECIE** | **HYDROGRAPHICS BASINS** | **AUTHOR** |
| --- | --- | --- | --- |
| MZ051339 | *Leporinus maculatus* | Maroni | Papa, Le Bail and Covain. 2021 |
| KF569001 | *Leporinus venerei* | Araguaia | Ramirez et al. 2016 |
| KY524536 | *Leporinus affinis* | Amazonas | Burns et al. 2017 |
| MF664228 | *Leporinus cf friderici* | Amazonas 2 | Silva-Santos et al. 2018 |
| MF664226 | *Leporinus cf friderici* | Mearim | Silva-Santos et al. 2018 |
| MZ051168 | *Leporinus friderici* | Tampok | Papa, Le Bail and Covain. 2021 |
| KF568982 | *Leporinus friderici* | Parana | Ramirez et al. 2016 |
| KM897440 | *Leporinus friderici* | Parana | Frantine-Silva et al. 2015 |
| HM906024 | *Leporinus piau* | Sao Francisco | Carvalho et al. 2011 |
| MF664285 | *Leporinus piau* | Jaguaribe | Silva-Santos et al. 2018 |
| FJ418763 | *Leporinus piau* | Amazonas | Ardura et al. 2010 |
| KF569000 | *Leporinus unitaeniatus* | Tocantins | Ramirez et al. 2016 |
| KF568986 | *Leporinus lacustris* | Tiete | Ramirez et al. 2016 |
| KF568985 | *Leporinus lacustris* | Cuiaba | Ramirez et al. 2016 |
| JN988998 | *Leporinus lacustris* | Parana | Pereira et al. 2013 |
| JN988997 | *Leporinus lacustris* | Parana | Pereira et al. 2013 |
| JN988996 | *Leporinus lacustris* | Parana | Pereira et al. 2013 |
| JN988995 | *Leporinus lacustris* | Parana | Pereira et al. 2013 |
| JN988994 | *Leporinus lacustris* | Parana | Pereira et al. 2013 |
| JN988993 | *Leporinus lacustris* | Parana | Pereira et al. 2013 |
| JN988992 | *Leporinus lacustris* | Parana | Pereira et al. 2013 |
| EU185566 | *Leporinus lacustris* | Parana | Santos et al. 2007, Unpublished data |
| HM405028 | *Megaleporinus elongatus* | Sao Francisco | Carvalho et al. 2011 |
| MN731315 | *Megaleporinus gaeiro* | Contas | Birindelli,Britskiand Ramirez. 2020 |
| KU134862 | *Megaleporinus* cf. *obtusidens* | Sao Francisco | Ramirez et al. 2016 |

**References**

**Ardura A, Linde AR, Moreira JC, Garcia-Vazquez E.** **2010**. DNA barcoding for conservation and management of Amazonian commercial fish. *Biological Conservation* 143:1438-1443 DOI 10.1016/j.biocon.2010.03.019.

**Birindelli JLO, Britski HA, Ramirez JL. 2020.** A new endangered species of Megaleporinus (Characiformes: Anostomidae) from the Rio de Contas basin, eastern Brazil. *Journal of Fish Biology* 96(6):1349-1359 DOI 10.1111/jfb.14299.

**Burns MD, Chatfield M, Birindelli JLO, Sidlauskas BL. 2017.** Systematic assessment of the Leporinus desmotes species complex, with a description of two new species. *Neotropical Ichthyology* 15(2):e160166 DOI 10.1590/1982-0224-20160166.

**Carvalho DC, Oliveira DA, Pompeu PS, Leal CG, Oliveira C, Hanne R. 2011.** Deep barcode divergence in Brazilian freshwater fishes: the case of the Sao Francisco River basin. *Mitochondrial DNA* 22(Supp 1):80-86 DOI 10.3109/19401736.2011.588214.

**Frantine-Silva W, Sofia SH, Orsi ML, Almeida FS. 2015.** DNA barcoding of freshwater ichthyoplankton in the Neotropics as a tool for ecological monitoring. *Molecular Ecology Resources* 15(5):1226-1237 DOI 10.1111/1755-0998.12385.

**Papa Y, Bail PYLe, Covain R. 2021.** Genetic landscape clustering of a large DNA barcoding data set reveals shared patterns of genetic divergence among freshwater fishes of the Maroni Basin. *Molecular Ecology Resources* 21(6):2109-2124 DOI 10.1111/1755-0998.13402.

**Pereira LH, Hanner R, Foresti F, Oliveira C. 2013**. Can DNA barcoding accurately discriminate megadiverse Neotropical freshwater fish fauna? *BMC Genomic Data* 14:20 DOI 10.1186/1471-2156-14-20.

**Ramirez JL, Carvalho-Costa LF, Venere PC, Carvalho DC, Troy WP, Galetti PM. 2016.** Testing monophyly of the freshwater fish Leporinus (Characiformes, Anostomidae) through molecular analysis. *Journal of Fish Biology* 88(3):1204-1214 DOI 10.1111/jfb.12906.

**Silva-Santos R, Ramirez JL, Freitas PD, Galetti Jr PM, Freitas PD. 2018**. Molecular Evidences of a Hidden Complex Scenario in Leporinus cf. friderici. *Frontiers in Genetics* 9:1-9 DOI 10.3389/fgene.2018.00047.
