# Supplementary material for: The DNA barcode reveals cryptic diversity and a new record for the genus Leporinus (Characiformes, Anostomidae) in the hydrographic basins of central northern Brazil
Source: PeerJ. 2023 May 25;11:e15184. doi: 10.7717/peerj.15184 (PMC10225125; doi:10.7717/peerj.15184)
Supplement: Table S4 — ML, Maximum Likelihood. Unnamed, group with more than one valid species. [file peerj-11-15184-s004.docx]

| Specimen |  |  | Nomenclature | Number of MOTUs: | | | | | ML arrangement |
| --- | --- | --- | --- | --- | --- | --- | --- | --- | --- |
|  |  |  |  | ABGD | ASAP | mPTP | bPTP | GMYC |  |
| LEP51 Leporinus piau Itapecuru |  |  | Unnamed 3 | 1 | 3 | 5 | 7 | 1 | Clade VI |
| LEP62 Leporinus piau Itapecuru | | |  | 1 | 3 | 5 | 7 | 1 |  |
| LEP68 Leporinus piau Itapecuru | | |  | 1 | 3 | 5 | 7 | 1 |  |
| LEP79 Leporinus piau Itapecuru |  |  |  | 1 | 3 | 5 | 7 | 1 |  |
| MF664226 Leporinus cf friderici Mearim |  |  |  | 1 | 3 | 5 | 7 | 1 |  |
| PER70 Leporinus piau Pericuma | | |  | 1 | 3 | 5 | 7 | 1 |  |
| PER71 Leporinus piau Pericuma |  |  |  | 1 | 3 | 5 | 7 | 1 |  |
| PER66 Leporinus piau Pericuma | | |  | 1 | 3 | 5 | 7 | 1 |  |
| PER67 Leporinus piau Pericuma |  |  |  | 1 | 3 | 5 | 7 | 1 |  |
| PER68 Leporinus piau Pericuma | | |  | 1 | 3 | 5 | 7 | 1 |  |
| PER69 Leporinus piau Pericuma |  |  |  | 1 | 3 | 5 | 7 | 1 |  |
| PER160 Leporinus piau Pericuma | | |  | 1 | 3 | 5 | 7 | 1 |  |
| PER161 Leporinus piau Pericuma |  |  |  | 1 | 3 | 5 | 7 | 1 |  |
| PER162 Leporinus piau Pericuma | | |  | 1 | 3 | 5 | 7 | 1 |  |
| PER163 Leporinus piau Pericuma |  |  |  | 1 | 3 | 5 | 7 | 1 |  |
| PER164 Leporinus piau Pericuma | | |  | 1 | 3 | 5 | 7 | 1 |  |
| LEP101 Leporinus piau Mearim |  |  |  | 1 | 3 | 5 | 7 | 1 |  |
| LEP143 Leporinus piau Mearim | |  |  | 1 | 3 | 5 | 7 | 1 |  |
| LEP144 Leporinus piau Mearim |  |  |  | 1 | 3 | 5 | 7 | 1 |  |
| LEP145 Leporinus piau Mearim | |  |  | 1 | 5 | 5 | 7 | 1 |  |
| LEP147 Leporinus piau MZUEL10530 Mearim |  |  |  | 1 | 3 | 5 | 7 | 1 |  |
| LEP148 Leporinus piau Mearim | |  |  | 1 | 3 | 5 | 7 | 1 |  |
| MEA55 Leporinus piau Mearim |  |  |  | 1 | 3 | 5 | 7 | 1 |  |
| MEA56 Leporinus piau Mearim | |  |  | 1 | 3 | 5 | 7 | 1 |  |
| MEA57 Leporinus piau Mearim |  |  |  | 1 | 3 | 5 | 7 | 1 |  |
| MEA59 Leporinus piau Mearim |  |  |  | 1 | 3 | 5 | 7 | 1 |  |
| MEA60 Leporinus piau Mearim | |  |  | 1 | 3 | 5 | 7 | 1 |  |
| MEA61 Leporinus piau Mearin |  |  |  | 1 | 3 | 5 | 7 | 1 |  |
| MEA63 Leporinus piau Mearim | |  |  | 1 | 3 | 5 | 7 | 1 |  |
| MEA64 Leporinus piau Mearim |  |  |  | 1 | 3 | 5 | 7 | 1 |  |
| MEA65 Leporinus piau Mearim | |  |  | 1 | 3 | 5 | 7 | 1 |  |
| MEA66 Leporinus piau Mearim |  |  |  | 1 | 3 | 5 | 7 | 1 |  |
| MEA99 Leporinus piau Mearim | |  |  | 1 | 3 | 5 | 7 | 1 |  |
| MEA100 Leporinus piau Mearim |  |  |  | 1 | 3 | 5 | 7 | 1 |  |
| MEA349 Leporinus piau Mearim | | |  | 1 | 3 | 5 | 7 | 1 |  |
| MEA350 Leporinus piau Mearim |  |  |  | 1 | 3 | 5 | 7 | 1 |  |
| MEA351 Leporinus piau Mearim | | |  | 1 | 3 | 5 | 7 | 1 |  |
| MEA493 Leporinus piau Mearim | | |  | 1 | 3 | 5 | 7 | 1 |  |
| MAE495 Leporinus piau Mearim |  |  |  | 1 | 3 | 5 | 7 | 1 |  |
| LEP112 Leporinus piau Mearim |  |  |  | 1 | 3 | 5 | 7 | 1 |  |
| LEP113 Leporinus piau Mearim | |  |  | 1 | 3 | 5 | 7 | 1 |  |
| LEP114 Leporinus piau Mearim |  |  |  | 1 | 3 | 5 | 7 | 1 |  |
| LEP116 Leporinus piau Mearim | |  |  | 1 | 3 | 5 | 7 | 1 |  |
| LEP119 Leporinus piau Mearim | |  |  | 1 | 3 | 5 | 7 | 1 |  |
| LEP120 Leporinus piau Mearim |  |  |  | 1 | 3 | 5 | 7 | 1 |  |
| LEP121 Leporinus piau Mearim | |  |  | 1 | 3 | 5 | 7 | 1 |  |
| LEP122 Leporinus piau Mearim |  |  |  | 1 | 3 | 5 | 7 | 1 |  |
| LEP123 Leporinus piau Mearim | |  |  | 1 | 3 | 5 | 7 | 1 |  |
| LEP124 Leporinus piau Mearim |  |  |  | 1 | 3 | 5 | 7 | 1 |  |
| LEP125 Leporinus piau Mearim | |  |  | 1 | 3 | 5 | 7 | 1 |  |
| LEP126 Leporinus piau Mearim |  |  |  | 1 | 3 | 5 | 7 | 1 |  |
| LEP127 Leporinus piau Mearim | |  |  | 1 | 3 | 5 | 7 | 1 |  |
| LEP129 Leporinus piau Mearim |  |  |  | 1 | 3 | 5 | 7 | 1 |  |
| LEP130 Leporinus piau Mearim | |  |  | 1 | 3 | 5 | 7 | 1 |  |
| LEP131 Leporinus piau MZUEL10531 Mearim |  |  |  | 1 | 3 | 5 | 7 | 1 |  |
| LEP132 Leporinus piau Mearim | |  |  | 1 | 3 | 5 | 7 | 1 |  |
| LEP133 Leporinus piau Mearim |  |  |  | 1 | 3 | 5 | 7 | 1 |  |
| LEP134 Leporinus piau Mearim | |  |  | 1 | 3 | 5 | 7 | 1 |  |
| LEP136 Leporinus piau Mearim |  |  |  | 1 | 3 | 5 | 7 | 1 |  |
| LEP137 Leporinus piau Mearim | |  |  | 1 | 3 | 5 | 7 | 1 |  |
| LEP138 Leporinus piau Mearim |  |  |  | 1 | 3 | 5 | 7 | 1 |  |
| LEP139 Leporinus piau Mearim | |  |  | 1 | 3 | 5 | 7 | 1 |  |
| LEP141 Leporinus piau Mearim |  |  |  | 1 | 5 | 5 | 7 | 1 |  |
| LEP142 Leporinus piau Mearim | |  |  | 1 | 3 | 5 | 7 | 1 |  |
| COR01 Leporinus piau Mearim |  |  |  | 1 | 3 | 5 | 7 | 1 |  |
| COR02 Leporinus piau Mearim | |  |  | 1 | 3 | 5 | 7 | 1 |  |
| COR03 Leporinus piau Mearim |  |  |  | 1 | 3 | 5 | 7 | 1 |  |
| COR04 Leporinus piau Mearim | |  |  | 1 | 3 | 5 | 7 | 1 |  |
| MF664285 Leporinus piau Jaguaribe | | | *Leporinus lacustris* | 1 | 11 | 5 | 7 | 1 |  |
| KF568986 Leporinus lacustris Tiete |  |  |  | 1 | 10 | 5 | 7 | 1 |  |
| KF568985 Leporinus lacustris Cuiaba | | |  | 1 | 10 | 5 | 7 | 1 |  |
| JN988998 Leporinus lacustris Parana |  |  |  | 1 | 10 | 5 | 7 | 1 |  |
| JN988997 Leporinus lacustris Parana | | |  | 1 | 10 | 5 | 7 | 1 |  |
| JN988996 Leporinus lacustris Parana |  |  |  | 1 | 10 | 5 | 7 | 1 |  |
| JN988995 Leporinus lacustris Parana | | |  | 1 | 10 | 5 | 7 | 1 |  |
| JN988994 Leporinus lacustris Parana |  |  |  | 1 | 10 | 5 | 7 | 1 |  |
| JN988993 Leporinus lacustris Parana | | |  | 1 | 10 | 5 | 7 | 1 |  |
| JN988992 Leporinus lacustris Parana |  |  |  | 1 | 10 | 5 | 7 | 1 |  |
| EU185566 Leporinus lacustris Parana | | |  | 1 | 10 | 5 | 7 | 1 |  |
| TUR35 Leporinus piau Turiacu | |  | Unnamed 2 | 1 | 3 | 5 | 7 | 1 |  |
| TUR36 Leporinus piau Turiacu |  |  |  | 1 | 3 | 5 | 7 | 1 |  |
| TUR37 Leporinus piau Turiacu | |  |  | 1 | 3 | 5 | 7 | 1 |  |
| TUR38 Leporinus piau Turiacu |  |  |  | 1 | 3 | 5 | 7 | 1 |  |
| TUR46 Leporinus piau Turiacu | |  |  | 1 | 3 | 5 | 7 | 1 |  |
| TUR256 Leporinus piau Turiacu |  |  |  | 1 | 3 | 5 | 7 | 1 |  |
| TUR257 Leporinus piau Turiacu | |  |  | 1 | 3 | 5 | 7 | 1 |  |
| TUR258 Leporinus piau Turiacu |  |  |  | 1 | 3 | 5 | 7 | 1 |  |
| TUR262 Leporinus piau Turiacu | |  |  | 1 | 3 | 5 | 7 | 1 |  |
| TUR263 Leporinus piau Turiacu |  |  |  | 1 | 3 | 5 | 7 | 1 |  |
| TUR264 Leporinus piau Turiacu | |  |  | 1 | 3 | 5 | 7 | 1 |  |
| MF664228 Leporinus cf friderici Amazonas2 |  |  |  | 1 | 3 | 5 | 7 | 1 |  |
| LEP01 Leporinus piau Itapecuru | | | *Leporinus venerei* | 1 | 1 | 5 | 7 | 1 |  |
| LEP02 Leporinus piau Itapecuru |  |  |  | 1 | 1 | 5 | 7 | 1 |  |
| LEP03 Leporinus piau Itapecuru | | |  | 1 | 1 | 5 | 7 | 1 |  |
| LEP05 Leporinus piau Itapecuru |  |  |  | 1 | 1 | 5 | 7 | 1 |  |
| LEP06 Leporinus piau Itapecuru | | |  | 1 | 1 | 5 | 7 | 1 |  |
| LEP07 Leporinus piau Itapecuru |  |  |  | 1 | 1 | 5 | 7 | 1 |  |
| LEP08 Leporinus piau Itapecuru | | |  | 1 | 1 | 5 | 7 | 1 |  |
| LEP11 Leporinus piau Itapecuru |  |  |  | 1 | 1 | 5 | 7 | 1 |  |
| LEP12 Leporinus piau Itapecuru | | |  | 1 | 1 | 5 | 7 | 1 |  |
| LEP15 Leporinus piau Itapecuru |  |  |  | 1 | 1 | 5 | 7 | 1 |  |
| LEP16 Leporinus piau Itapecuru | | |  | 1 | 1 | 5 | 7 | 1 |  |
| LEP17 Leporinus piau Itapecuru |  |  |  | 1 | 1 | 5 | 7 | 1 |  |
| LEP18 Leporinus piau Itapecuru | | |  | 1 | 1 | 5 | 7 | 1 |  |
| LEP19 Leporinus piau Itapecuru |  |  |  | 1 | 1 | 5 | 7 | 1 |  |
| LEP20 Leporinus piau Itapecuru | | |  | 1 | 1 | 5 | 7 | 1 |  |
| LEP21 Leporinus piau Itapecuru |  |  |  | 1 | 1 | 5 | 7 | 1 |  |
| LEP23 Leporinus piau Itapecuru | | |  | 1 | 1 | 5 | 7 | 1 |  |
| LEP25 Leporinus piau Itapecuru |  |  |  | 1 | 1 | 5 | 7 | 1 |  |
| LEP29 Leporinus piau Itapecuru | | |  | 1 | 1 | 5 | 7 | 1 |  |
| LEP28 Leporinus piau Itapecuru |  |  |  | 1 | 1 | 5 | 7 | 1 |  |
| LEP30 Leporinus piau Itapecuru | | |  | 1 | 1 | 5 | 7 | 1 |  |
| LEP45 Leporinus piau Itapecuru |  |  |  | 1 | 1 | 5 | 7 | 1 |  |
| LEP46 Leporinus piau Itapecuru | | |  | 1 | 1 | 5 | 7 | 1 |  |
| LEP47 Leporinus piau Itapecuru |  |  |  | 1 | 1 | 5 | 7 | 1 |  |
| LEP48 Leporinus piau Itapecuru | | |  | 1 | 1 | 5 | 7 | 1 |  |
| LEP49 Leporinus piau Itapecuru |  |  |  | 1 | 1 | 5 | 7 | 1 |  |
| LEP50 Leporinus piau Itapecuru | | |  | 1 | 1 | 5 | 7 | 1 |  |
| LEP53 Leporinus piau Itapecuru | | |  | 1 | 1 | 5 | 7 | 1 |  |
| LEP78 Leporinus piau Itapecuru |  |  |  | 1 | 1 | 5 | 7 | 1 |  |
| LEP67 Leporinus piau Itapecuru |  |  |  | 1 | 12 | 5 | 7 | 1 |  |
| LEP80 Leporinus piau Itapecuru | | |  | 1 | 1 | 5 | 7 | 1 |  |
| MEA58 Leporinus piau Mearim | |  |  | 1 | 1 | 5 | 7 | 1 |  |
| MEA352 Leporinus piau Mearim |  |  |  | 1 | 1 | 5 | 7 | 1 |  |
| MEA353 Leporinus piau Mearim | | |  | 1 | 1 | 5 | 7 | 1 |  |
| MEA492 Leporinus piau Mearim |  |  |  | 1 | 1 | 5 | 7 | 1 |  |
| MEA494 Leporinus piau Mearim | | |  | 1 | 1 | 5 | 7 | 1 |  |
| MEA496 Leporinus piau Mearim |  |  |  | 1 | 1 | 5 | 7 | 1 |  |
| MEA497 Leporinus piau Mearim | | |  | 1 | 1 | 5 | 7 | 1 |  |
| LEP115 Leporinus piau Mearim |  |  |  | 1 | 1 | 5 | 7 | 1 |  |
| COR05 Leporinus piau Mearim |  |  |  | 1 | 1 | 5 | 7 | 1 |  |
| TUR34 Leporinus piau Turiacu |  |  |  | 1 | 2 | 5 | 7 | 1 |  |
| PERI01 Leporinus piau Peria | |  |  | 1 | 1 | 5 | 7 | 1 |  |
| PERI02 Leporinus piau Peria |  |  |  | 1 | 1 | 5 | 7 | 1 |  |
| PRE01 Leporinus piau Preguicas | | |  | 1 | 1 | 5 | 7 | 1 |  |
| TO495 Leporinus venerei Tocantins |  |  |  | 1 | 1 | 5 | 7 | 1 |  |
| TO493 Leporinus venerei Tocantins | | |  | 1 | 1 | 5 | 7 | 1 |  |
| TO496 Leporinus venerei Tocantins |  |  |  | 1 | 1 | 5 | 7 | 1 |  |
| TO548 Leporinus venerei Tocantins | | |  | 1 | 1 | 5 | 7 | 1 |  |
| KF569001 Leporinus venerei Araguaia |  |  |  | 1 | 1 | 5 | 7 | 1 |  |
| PALEP03 Leporinus piau Parnaiba | | | Unnamed 1 | 2 | 4 | 8 | 5 | 2 | Clade V |
| PALEP05 Leporinus piau Parnaiba |  |  |  | 2 | 4 | 8 | 5 | 2 |  |
| LEP98 Leporinus piau MZUSP110830 Mearim | | |  | 2 | 4 | 8 | 5 | 2 |  |
| FJ418763 Leporinus piau Amazonas |  |  |  | 2 | 4 | 8 | 5 | 2 |  |
| HM906024 leporinus piau Sao Francisco | | |  | 2 | 4 | 8 | 5 | 2 |  |
| KM897440 Leporinus friderici Parana |  |  |  | 2 | 4 | 8 | 5 | 2 |  |
| MZ051168 Leporinus friderici Tampok | | |  | 8 | 13 | 9 | 6 | 2 |  |
| KF568982 Leporinus friderici Parana |  |  |  | 2 | 4 | 8 | 5 | 2 |  |
| TO640 Leporinus affinis Tocantins | | | *Leporinus affinis* | 5 | 7 | 7 | 9 | 3 | Clade II |
| TO661 Leporinus affinis Tocantins |  |  |  | 5 | 7 | 7 | 9 | 3 |  |
| TO603 Leporinus affinis Tocantins | | |  | 5 | 7 | 7 | 9 | 3 |  |
| TO634 Leporinus affinis Tocantins |  |  |  | 5 | 7 | 7 | 9 | 3 |  |
| TO636 Leporinus affinis Tocantins | | |  | 5 | 7 | 7 | 9 | 3 |  |
| TO637 Leporinus affinis Tocantins |  |  |  | 5 | 7 | 7 | 9 | 3 |  |
| TO638 Leporinus affinis Tocantins | | |  | 5 | 7 | 7 | 9 | 3 |  |
| KY524536 Leporinus affinis Amazonas |  |  |  | 10 | 15 | 6 | 8 | 3 |  |
| TO599 Leporinus unitaeniatus Tocantins | | | *Leporinus unitaeniatus* | 6 | 8 | 2 | 2 | 4 | Clade III |
| TO600 Leporinus unitaeniatus Tocantins |  |  |  | 6 | 8 | 2 | 2 | 4 |  |
| TO601 Leporinus unitaeniatus Tocantins | | |  | 6 | 8 | 2 | 2 | 4 |  |
| TO602 Leporinus unitaeniatus Tocantins |  |  |  | 6 | 8 | 2 | 2 | 4 |  |
| KF569000 Leporinus unitaeniatus Tocantins | | |  | 6 | 8 | 2 | 2 | 4 |  |
| TO557 Leporinus maculatus Tocantins |  |  | *Leporinus maculatus* | 4 | 6 | 3 | 3 | 5 | Clade IV |
| MZ051339 Leporinus maculatus Maroni | | |  | 9 | 14 | 4 | 4 | 5 |  |
| PALEP01 Leporinus piau Parnaiba |  |  | *Megaleporinus* sp. | 7 | 9 | 1 | 1 | 6 | Clade I |
| PALEP04 Leporinus piau Parnaiba | | |  | 7 | 9 | 1 | 1 | 6 |  |
| PALEP06 Leporinus piau Parnaiba |  |  |  | 7 | 9 | 1 | 1 | 6 |  |
| PALEP08 Leporinus piau Parnaiba | | |  | 7 | 9 | 1 | 1 | 6 |  |
| PALEP09 Leporinus piau Parnaiba |  |  |  | 7 | 9 | 1 | 1 | 6 |  |
| PALEP13 Leporinus piau Parnaiba | | |  | 7 | 9 | 1 | 1 | 6 |  |
| PALEP14 Leporinus piau Parnaiba |  |  |  | 7 | 9 | 1 | 1 | 6 |  |
| PALEP15 Leporinus piau Parnaiba | | |  | 7 | 9 | 1 | 1 | 6 |  |
| PALEP16 Leporinus piau Parnaiba |  |  |  | 7 | 9 | 1 | 1 | 6 |  |
| PALEP18 Leporinus piau Parnaiba | | |  | 7 | 9 | 1 | 1 | 6 |  |
| PALEP19 Leporinus piau Parnaiba |  |  |  | 7 | 9 | 1 | 1 | 6 |  |
| PALEP20 Leporinus piau Parnaiba | | |  | 7 | 9 | 1 | 1 | 6 |  |
| PALEP21 Leporinus piau Parnaiba |  |  |  | 7 | 9 | 1 | 1 | 6 |  |
| PALEP22 Leporinus piau Parnaiba | | |  | 7 | 9 | 1 | 1 | 6 |  |
| PALEP23 Leporinus piau Parnaiba |  |  |  | 7 | 9 | 1 | 1 | 6 |  |
| PALEP25 Leporinus piau Parnaiba | | |  | 7 | 9 | 1 | 1 | 6 |  |
| HM405028 Megaleporinus elongatus Sao Francisco |  |  |  | 11 | 9 | 1 | 1 | 6 |  |
| KU134862 Megaleporinus cf. obtusidens Sao Francisco | | | | 10 | 9 | 1 | 1 | 6 |  |
| MN731315 Megaleporinus gaieiro Contas |  |  |  | 12 | 9 | 1 | 1 | 6 |  |
